# COVID-19: a crisis of the female self-employed

**DOI:** 10.1007/s00148-021-00849-y

**Published:** 2021-06-11

**Authors:** Daniel Graeber, Alexander S. Kritikos, Johannes Seebauer

**Affiliations:** 1 DIW Berlin, Mohrenstr. 58, 10117 Berlin, Germany; 2 University of Potsdam, Potsdam, Germany; 3 GLO, Essen, Germany; 4 Freie Universität Berlin, Berlin, Germany

**Keywords:** Self-employed, COVID-19, Income, Gender, Representative real-time survey data, Decomposition methods, J16, L26, J31, J71, I18

## Abstract

We investigate how the economic consequences of the pandemic and the government-mandated measures to contain its spread affect the self-employed — particularly women — in Germany. For our analysis, we use representative, real-time survey data in which respondents were asked about their situation during the COVID-19 pandemic. Our findings indicate that among the self-employed, who generally face a higher likelihood of income losses due to COVID-19 than employees, women are about one-third more likely to experience income losses than their male counterparts. We do not find a comparable gender gap among employees. Our results further suggest that the gender gap among the self-employed is largely explained by the fact that women disproportionately work in industries that are more severely affected by the COVID-19 pandemic. Our analysis of potential mechanisms reveals that women are significantly more likely to be impacted by government-imposed restrictions, e.g., the regulation of opening hours. We conclude that future policy measures intending to mitigate the consequences of such shocks should account for this considerable variation in economic hardship.

## Introduction

The unprecedented shutdown of businesses in specific industries, social distancing guidelines, and overall insecurity caused by the COVID-19 pandemic resulted in the temporary halt of major parts of the economy in many countries in 2020, with dire consequences for these economies (Milani 2021). The service sector, which often necessitates physical proximity, was particularly affected (Barbieri et al. 2020). At the same time, the service sector depends more on self-employed individuals than the manufacturing sector, where the vast majority of workers are employees. In particular, self-employed women are more likely to work in service industries than self-employed men: According to the OECD (2017), 91% of self-employed women and 68% of self-employed men in Germany worked in the service sector in 2016.

The COVID-19 pandemic initiated a public debate as to what extent the female working population experienced greater income and employment reductions. This is particularly relevant since women are often the primary caregivers in the family and, as such, were also confronted with the closure of schools and daycare centers (Alon et al. 2020). However, the debate revolving around the gender gap and the impact of the COVID-19 pandemic does not, thus far, differentiate between forms of employment, although initial descriptive evidence points to stronger negative effects for self-employed women (see, e.g., Ifo Institute and forsa (2020) for Germany and Kalenkoski and Pabilonia (2020) for the USA). In this paper, we investigate whether women in self-employment and among employees are more severely affected by the economic consequences of the COVID-19 pandemic and associated non-pharmaceutical interventions (NPI) than men. To the best of our knowledge, we are the first to explicitly contrast the experience of the self-employed with employees during the COVID-19 pandemic and, by doing so, to identify where gender disparities occurred as a consequence of the pandemic.

The particular focus on self-employed individuals is warranted by the increasing importance of self-employment and entrepreneurship for modern economies. For example, in Germany, around 4.2 million individuals — about ten percent of the working population — are self-employed, running diverse businesses either without or with additional employees, often micro-businesses with up to 10 employees. In sum, the self-employed contribute substantially to the economic development in Germany (Audretsch et al. 2020). It is further important to note that, while there is still a significant gender gap among the self-employed, the share of women has been increasing steadily since the turn of the century (Fritsch et al. 2015).

Our study proceeds in three steps. First, we contextualize our analysis on the comparison between female and male workers in both forms of employment by investigating the differential impact of the COVID-19 pandemic on the self-employed and employees. Second, in our main analysis, we examine the gender gap in the effect of the pandemic on labor market outcomes, focusing on the self-employed. Third, we provide evidence on potential mechanisms driving the observed gender differences among the self-employed. For our analysis, we use the Socio-Economic Panel-CoV (SOEP-CoV), a novel data set sufficiently rich to allow for such a comparison, as it enables us to control for individual-level heterogeneity to a large extent. SOEP-CoV surveyed a randomly selected subset of respondents from the SOEP who were asked to answer a wide array of questions about their economic situation, family situation, health, the use of public support instruments, as well as attitudes during the early stages of the COVID-19 pandemic. The SOEP is a representative household panel in Germany that surveys respondents annually since 1984 (Goebel et al. 2019). By design, the SOEP-CoV enables us to link individual respondents to their pre-crisis information. Thus, we can exploit rich information on the respondents, including their pre-crisis household income, education, household characteristics, personality traits, and employment experience, among others. Therefore, we are able to analyze whether individual characteristics that are known to be important determinants of self-employment influence outcomes during the COVID-19 pandemic (see, e.g., Parker 2018).

With this data at hand, we perform multivariate analyses, first comparing the gap in labor market outcomes between employed and self-employed respondents. We show that there are significant differences in the influence of the COVID-19 pandemic and associated NPIs on the two employment forms: The self-employed are about 42 percentage points more likely to report losses of gross income than employees and 30 percentage points more likely to report a reduction in working hours. Turning to gender differences in the influence of the COVID-19 pandemic, we find that self-employed women are about one-third more likely than self-employed men to experience income losses due to the COVID-19 pandemic. We do not find a comparable gender gap among employees.

We then decompose the gender gap in the probability of income losses among the self-employed using the Gelbach decomposition (Gelbach 2016), allowing us to decompose different sets of covariates into their individual contribution to the gender gap. We show that the gender gaps in the probability of income losses and reductions in working hours due to the COVID-19 pandemic are driven by the fact that self-employed women are disproportionately active in industries that are more severely affected by the COVID-19 pandemic. We do not find such evidence for employees.

Lastly, we provide evidence for a likely channel driving the gender gap among the self-employed. We find that self-employed women are 20 percentage points more likely to be affected by regulations adopted during the COVID-19 pandemic.

We show that our results are, once again, driven by the disproportionate sorting of self-employed women into industries that were more severely restricted by the NPIs implemented. Moreover, we present evidence that these restrictions mediate the relationship between industry-sorting and income losses. We also find suggestive evidence that gendered household production contributes to the gender gap in income losses. However, this effect is of second order compared to the contribution of the industry affiliation.

We contribute to the literature in several ways. First, we contrast the gender gap between employees and self-employed individuals in the labor market during the early onset of the COVID-19 pandemic. In contrast to related studies relying on the U.S. Current Population Survey (Fairlie 2020; Kalenkoski and Pabilonia 2020) or the Canadian Labour Force Survey (Beland et al. 2020), the SOEP-CoV contains information on earnings losses due to the COVID-19 pandemic. Adams-Prassl et al. (2020), who collected their own data, are a notable exception in that they do have information on earnings losses. They do not find gender differences in realized job or earnings losses for Germany. While Adams-Prassl et al. (2020) provide important initial evidence, they do not distinguish between self-employed individuals and employees with respect to the gender gap. This is an important distinction since the labor market in Germany is characterized by stronger rigidities than other countries, limiting the extent to which firms can cut the wages of their employees (e.g., Burda 2016). Furthermore, policy measures taken by the federal government were mostly aimed at stabilizing the earnings and employment trajectories of employees. By contrast, self-employed individuals, as residual claimants, are more vulnerable to economic shocks like the COVID-19 pandemic.

Second, we contribute to the broader literature on gender gaps in labor markets (e.g., Blau and Kahn 2017; Goldin et al. 2017; Meara et al. 2020) documenting earnings gaps, which the authors, among other factors, attribute to the selection of women into occupations or sectors that are associated with lower average wages. We complement this literature with our finding that the disproportionate representation of women in certain industries also translates into a gender gap in the impact of the COVID-19 pandemic. Third, our finding that government-mandated regulations are an important driver of the gender gap in the impact of the pandemic on the self-employed constitutes novel evidence in the literature.

Lastly, we also contribute to a strand of literature studying the consequences of the spread of communicable diseases on economic well-being in general (e.g., Karlsson et al. 2014; Barro et al. 2020; Correia et al. 2020; Velde 2020). These studies mainly investigate the impact of the 1918 Spanish flu. While providing important insights, these studies are hampered by limited data due to the historical nature of the event. In this context, our finding that NPIs have unintended consequences for gender equality implies that this variation in economic suffering needs to be accounted for when addressing the ongoing COVID-19 pandemic or any future public health crisis involving communicable diseases of a similar or even greater magnitude.

## Background: the COVID-19 pandemic, policy measures, and female self-employment

In this section, we provide a short summary of policy measures enacted in Germany in the early months of the pandemic, before we relate our study to contemporaneous research on the impact of COVID-19 on self-employment, as well as on the gender gap in self-employment.

### Policy measures in the wake of the COVID-19 pandemic

In order to contain SARS-CoV-2, the German government imposed strong restrictions beginning in March 2020, shortly before our period of observation. These NPIs included the closure of schools, daycare centers, restaurants, service companies in the field of personal hygiene, and most shops — with exceptions for grocery stores. All public events were canceled and travel was restricted. Meetings in public were limited to two individuals, while people were required to keep a minimum distance of 1.5 meters from other people in public spaces (Federal Ministry of Health 2020). While these measures were certainly sensible from an epidemiological perspective (e.g., Qiu et al. 2020, Bonacini et al. 2021), more than half of the self-employed experienced sales and income losses in spring 2020 (Kritikos et al. 2020).

The German government introduced several economic policy measures to mitigate the economic consequences of the COVID-19 pandemic. The most prominent policy measure being the expansion of “*Kurzarbeit*,” the established short-time work compensation scheme where the employment agency covers up to 67% of employees’ net income.^1^ As the self-employed are not covered by this instrument, the federal government released an emergency aid package of up to €50 billion for the self-employed. This program supported the self-employed facing strong losses in revenues with lump sum payments of up to €15,000. The use of this payment was limited to covering fixed operating costs and temporarily increased the subjective survival probability (Block et al. 2020). In addition, the self-employed received easier access to unemployment benefits “*Arbeitslosengeld 2*” (Federal Ministry for Economic Affairs and Energy 2020).

### Related research on self-employment

Crisis-related research on self-employment has received considerable attention (see, e.g., Doern et al. 2019). On the one hand, a large part of this literature focuses on the question of how individuals decide about venturing new businesses in reaction to a crisis (see, e.g., Siemer 2014) and, on the other hand, the crisis management of existing businesses (see, e.g., Davidsson and Gordon 2016). Much less is known about the magnitude of the impact of crises on the self-employed; existing research is often based on qualitative interviews with retrospective questions (see, e.g., Doern^2016^).

In contrast to other crises, the COVID-19 pandemic affects nearly the entire self-employed population, as is documented in contemporaneous research, all of which shows that self-employed individuals suffered significantly from the consequences of the COVID-19 pandemic.^2^ For the UK, Blundell and Machin (2020) show that three out of four self-employed individuals report a reduced work load. While they provide important evidence on the impact of the COVID-19 pandemic on self-employed individuals, they do not consider gender differences in their analysis. Fairlie (2020) documents that the activity of business owners in the USA plummeted by 3.3 million, or 22%, during the early stages of the COVID-19 pandemic. Fairlie (2020) also documents considerable race and gender differences in the effects of the COVID-19 pandemic on the number of active small businesses. In contrast to our study, Fairlie (2020) does not have information on income losses. Kalenkoski and Pabilonia (2020), who focus on unincorporated self-employed in the USA, find that self-employed individuals are about 57 percentage points less likely to be employed in April 2020, compared to February. The authors, like Fairlie (2020), also do not have information on income. Kalenkoski and Pabilonia (2020) likewise document gender differences in the effects of the COVID-19 pandemic on self-employed individuals. Lastly, Beland et al. (2020) report an activity decline of 14.8% for incorporated and 10.1% for unincorporated entities in Canada. They also find gender differences in the impact on COVID-19 on employment and hours, yet do not analyze this differential impact, nor do they have information on income.

In summary, we expand the analysis of gender differences in the effect of the COVID-19 pandemic on self-employed individuals in two important ways: First, we have information on income losses, in addition to income information from 2019. Second, we provide important evidence that it is the sorting of women into industries that are more strongly affected by the pandemic and associated NPIs that drives the observed gender differences among the self-employed.

Lastly, our study also relates to the literature on gender gaps in self-employment. In most countries, fewer women than men are self-employed (Elam et al. 2019). While the share of women in self-employment was as low as 25% at the turn of the century in Germany (Fritsch et al. 2015), it increased continously to nearly 35% in 2017 (Günther and Marder-Puch 2019). This development was also aided by the active promotion of self-employment via start-up subsidies (see, e.g., Caliendo and Künn 2015). The literature documents a variety of reasons for the still existing gender gap in self-employment, ranging from differences in the intergenerational transfer of human capital (see, e.g., Georgellis and Wall 2005), differing influences of age (see, e.g., Leoni and Falk 2010), differing risk attitudes (Caliendo et al. 2014), self-confidence (see, e.g., Koellinger et al. 2013), or the willingness to compete (see, e.g., Bönte and Piegeler 2013), while there is also substantial heterogeneity in employment decisions both among women and between women and men (Patrick et al. 2016). Certainly, these differences may inform the implications of our findings for the development of female self-employment post-pandemic.

## Data

In this section we briefly describe our data set and discuss the outcome variables used in the analysis. We then provide descriptive statistics of these outcome variables.

### SOEP-CoV

For our analysis, we use a unique data source to estimate the effect of the COVID-19 pandemic on the self-employed. The SOEP-CoV survey was launched in April 2020 to investigate the socio-economic consequences of the COVID-19 pandemic in Germany. In the first part of this special survey, respondents, interviewed in nine waves between April and July 2020, were asked about their economic status, family situation, health information, and attitudes during the COVID-19 pandemic (Kühne et al. 2020). Importantly, the SOEP-CoV questionnaire includes a set of questions targeting self-employed individuals.

What makes the SOEP-CoV particularly useful, is its integration into the SOEP. The Socio-Economic Panel (SOEP) is a representative, longitudinal survey of households in Germany that started 1984 and is administered to households and the households’ members on a yearly basis since then.^3^ As of 2020, the SOEP includes approximately 20,000 households with more than 30,000 adult household members. The SOEP contains information on the households and its members’ economic situation, education, and attitudes, among other things (Goebel et al. 2019).

The respondents surveyed in the SOEP-CoV are a random subset of the SOEP population. Thus, it combines the wealth of longitudinal, pre-pandemic information from the SOEP with a wide array of questions that are related specifically to the COVID-19 pandemic. These unique features make the SOEP-CoV the ideal data set to analyze our research questions. For our analysis, we focus on individuals who are either gainfully employed (part- and full-time) or self-employed. We do not consider self-employed individuals who identified as helping family members in 2019.

### Outcome variables

In our analysis, we investigate the differential influence of the COVID-19 pandemic by self-employment status and gender. We focus on the likelihood of experiencing a decrease in income (gross earnings), working hours, and working at least partially from home due to the COVID-19 pandemic. In addition, we also have information on the magnitude of losses of monthly income and reductions in weekly working hours. These outcomes jointly determine how individuals have experienced the COVID-19 crisis to a significant degree and allow for examining differences between employees and the self-employed. Importantly, the questions on income losses, reductions in working hours, and remote work are framed causally. That is, respondents are explicitly asked whether, and to what extent, income and hours worked have changed due to the pandemic. Similarly, they are asked whether they are working from home due to the pandemic, either in part or completely.

While employees are partially protected from income losses in the short-run, when they have fixed employment contracts, this does not apply to the self-employed. The main mechanisms through which employees can face changes in income and working hours are job losses and participation of their employer in short-time work schemes. Furthermore, employees and self-employed individuals may select into different industries. To the extent that these industries are hit by the crisis to varying degrees, the likelihood of reductions in incomes and working hours will differ. The same argument applies to gender differences. To the extent that women select into different industries and occupations than men, along with the extent to which these are differently affected by the pandemic, its effect on income and hours will be different. Finally, the potential for working remotely vastly differs across sectors and jobs (von Gaudecker et al. 2020; Alipour et al. 2020; Dingel and Neiman 2020). While front-line workers continue to be potentially exposed to the virus throughout the pandemic, it is more easily possible for individuals in office jobs to do their work partly, if not completely, from home. By contrast, the arts and entertainment industry, where remote work is nearly non-existent, came to an almost complete halt. Thus, in our main analysis, we shed light on the heterogeneous influence of the COVID-19 pandemic on these core outcomes, which shape the experience of the workforce during the COVID-19 pandemic. Other variables used in the analysis are described in Table 5 in Appendix 1.

### Descriptive statistics on outcomes at the extensive margin

**Fig. 1:**
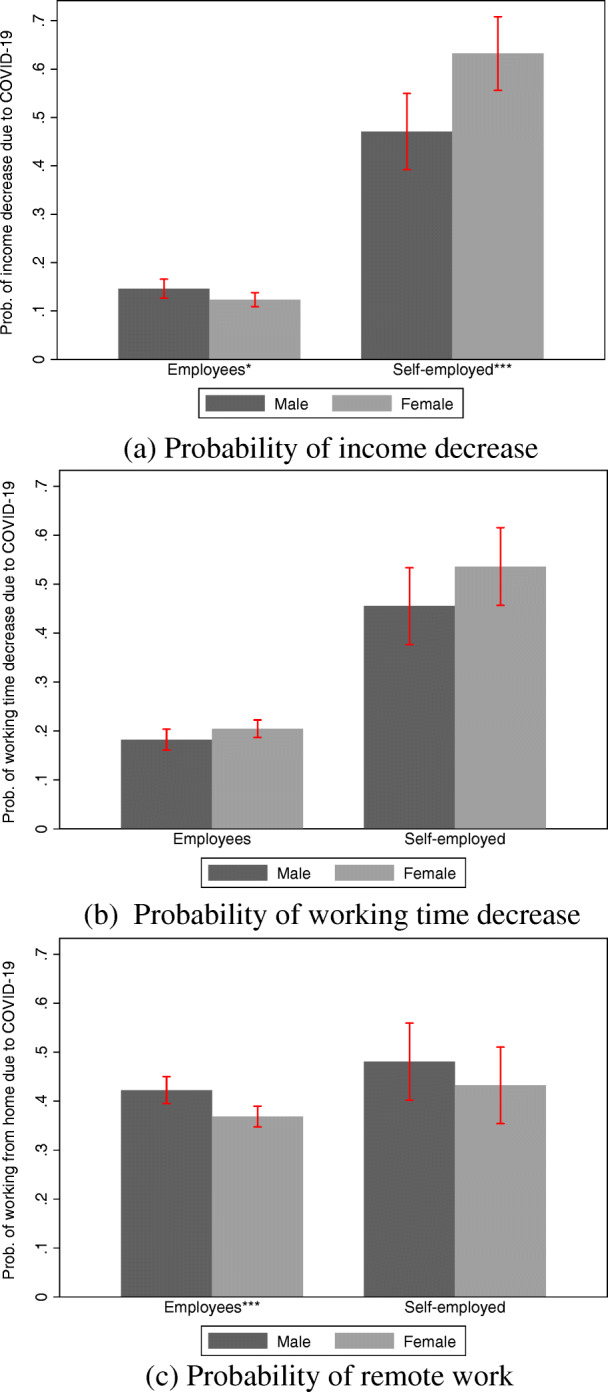
Gender comparison of raw differences in probabilities of labor market outcomes. Note: **a**–**c** display the raw differences in the probability of labor market outcomes over employment status and gender, respectively. Vertical bars correspond to 95% confidence intervals. The stars next to the respective employment group indicate whether the mean differences by gender within the groups are statistically significant and read *p < 0.10, **p < 0.05, ***p < 0.01. Details are displayed in Tables 6, 7, and 8 in Appendix 1

Tables 6, 7, and 8 in Appendix 1 show summary statistics for our analysis sample. The sample is restricted to those individuals for whom the full set of control variables used is available. Importantly, they describe how self-employed individuals were affected by the pandemic in comparison to employees with respect to our outcomes of interest, and how these experiences differ by gender in both employment forms. Figure 1a–c illustrate these differences. The probability of facing reductions in income and working hours is considerably larger among the self-employed than among employees. Around 55% of self-employed individuals report a decline in income and around 50% in working hours, while this is the case for only 13% of employees with respect to income and 20% of them with respect to working hours. A drop in demand directly affects the income and workload of self-employed individuals, whereas income and working hours of employees are affected by a sales decrease in their firms only if they are sent into short-time work or laid off. While job losses following the initial COVID-19 pandemic lockdown are rare in Germany, at least when compared to the experience of other countries (Adams-Prassl et al. 2020), the instrument of short-time work is used extensively.^4^ Although the difference is notably smaller, remote work as a direct consequence of the pandemic is also more common among the self-employed (with 46%) than among employees (39%).

Figure 1 also shows striking patterns of gender differences in the outcome variables. Most notably, there is a significant gender gap within the group of self-employed individuals: 63% of self-employed women faced income losses as opposed to 47% of their male counterparts. At the same time, 54% of self-employed women and 46% of self-employed men reduced their working hours. With respect to remote work, the gender gap is smaller and, in fact, inverts with men being more likely to work from home than women.

These gender gaps, however, are not replicated among employees. Here, the gender difference in the probability of income losses amounts to roughly two percentage points and inverts. The gender gap in the probability of working from home is similar in magnitude to that of the self-employed. Thus, there is a significant self-employment gap in the outcomes of interest with sizeable gender differences that are concentrated among the self-employed.

### Descriptive statistics on decreases in income and hours at the intensive margin

**Fig. 2:**
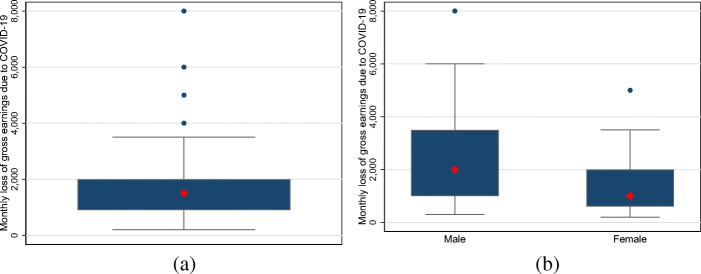
The distributions of absolute monthly losses in gross earnings among self-employed individuals. Note: **a** and **b** display boxplots for monthly income losses among all self-employed individuals as well as self-employed men and women. The large red diamond indicates the median. The upper and lower ends of the box display the range between the 25th and 75th percentiles. The whiskers span all data points within 1.5 inter-quartile range of the nearer quartile. Small blue dots indicate observations outside the whiskers. **a** All. **b** Gender differences

We also provide descriptive evidence on the magnitude of decreases in income and working hours among the self-employed, beginning with the magnitude of losses in monthly earnings.^5^ Figure 2 displays the boxplots for monthly absolute income losses for all self-employed individuals as well as separately for women and men. The median and mean of monthly income losses due to the COVID-19 pandemic are €1500 and €3021 for all self-employed individuals, respectively. Self-employed men experience higher absolute income losses, with median income losses of €2000, compared to €1000 for women. The corresponding means are €4741 and €1945 for self-employed men and women, respectively.

To measure relative losses, we relate the magnitude of income losses to 2019 earnings by dividing the absolute monthly losses in gross earnings by the monthly gross earnings of the previous year. However, since intra-year changes in income are frequent among the self-employed, the following results should be interpreted with some caution.^6^

**Fig. 3:**
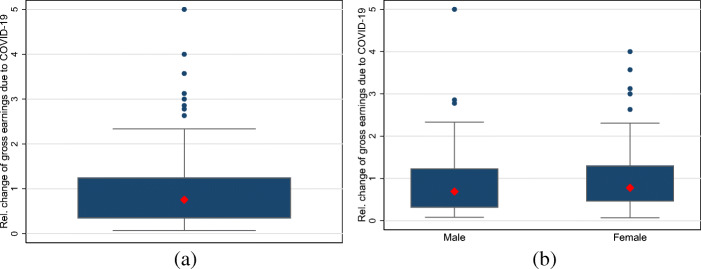
The distributions of monthly relative losses in income (gross earnings) among self-employed individuals. Note: **a** and **b** display boxplots for relative monthly income losses among all self-employed individuals as well as self-employed men and women. The large red diamond indicates the median. The upper and lower end of the box display the range between the 25th and 75th percentiles. The whiskers span all data points within 1.5 inter-quartile range of the nearer quartile. Small blue dots indicate observations outside the whiskers. **a** All. **b** Gender differences

The results for relative income losses are shown in Fig. 3. Figure 3a displays the boxplot for all self-employed individuals. The median and mean of relative income losses among all self-employed individuals are 0.77 and 1.54, respectively. Figure 3b displays the boxplot for self-employed men and women. The median is 0.79 for self-employed women and 0.69 for men. Thus, in contrast to absolute losses, this suggests that the relative income losses tend to be larger for women. However, a formal median comparison indicates that we cannot reject equality of medians for self-employed men and women.

**Fig. 4:**
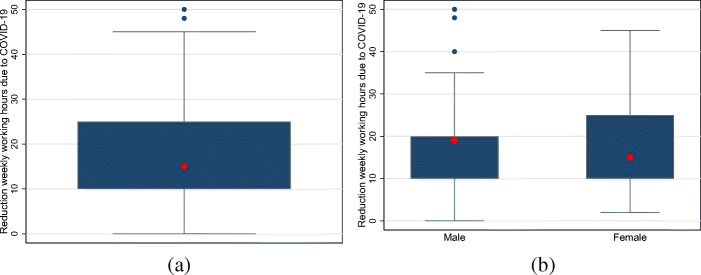
The distributions of the reduction in weekly working hours among the self-employed. Note: **a** and **b** display boxplots for reductions in weekly working hours among all self-employed individuals as well as self-employed men and women. The large red diamond indicates the median. The upper and lower end of the box display the range between the 25th and 75th percentiles. The whiskers span all data points within 1.5 inter-quartile range of the nearer quartile. Small blue dots indicate observations outside the whiskers. **a** All. **b** Gender differences

Turning to the reduction of weekly working hours due to COVID-19, we find that the median and mean absolute decreases are 15 and 18.07 hrs, respectively.^7^ The corresponding distribution is displayed in Fig. 4a. Figure 4b shows that the median and mean reduction of working hours for self-employed men are 19 and 18.60 hrs, respectively. The corresponding figures for self-employed women are slightly smaller, with a median of 15 and a mean of 17.61 hrs. Yet again, formal tests of equality across groups do not allow us to reject the hypothesis of no differences.

**Fig. 5:**
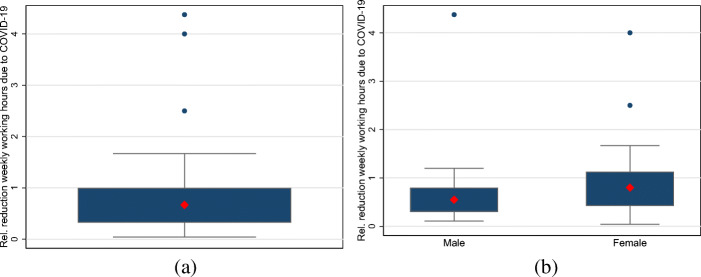
The distributions of relative reductions in weekly working hours among the self-employed. Note: **a** and **b** display boxplots for relative reductions in weekly working hours among all self-employed individuals as well as self-employed men and women. The large red diamond indicates the median. The upper and lower end of the box display the range between the 25th and 75th percentiles. The whiskers span all data points within 1.5 inter-quartile range of the nearer quartile. Small blue dots indicate observations outside the whiskers. **a** All. **b** Gender differences

Lastly, we focus on relative reductions in weekly working hours. We divide the decrease in weekly working hours due to COVID-19 by the actual weekly working hours of the previous year. The distributions are depicted in Fig. 5.^8^ Figure 5a displays the respective distribution for all self-employed individuals. The median and mean are 0.6 and 0.78, respectively. Figure 5b displays the corresponding gender-specific distributions. For self-employed men, the median and mean of relative working hours reductions are 0.5 and 0.77. For self-employed women, these figures are 0.63 and 0.79, respectively. Once again, the differences between men and women are not statistically significant.

## Multivariate analysis

Our descriptive results in the previous section show that the crisis following the COVID-19 pandemic impacts the female self-employed considerably more than all other groups. In this section, we perform multivariate analyses to better understand how these differences emerge.

### Comparison of the self-employed and employees

To put the analysis of the gender gap among the self-employed into a larger context, we start with a comparison of all self-employed individuals with employees. Table 1 shows the results of a regression of indicators for a decrease in income, a decrease in working hours, and working from home, respectively, on an indicator for self-employment. While the odd-numbered columns only include state indicators as well as week indicators, the even columns expand the set of controls to include our complete set of controls.^9^ With only state and week fixed effects as controls, self-employed individuals are 42 percentage points more likely to have experienced an income loss and 30 percentage points more likely to have experienced a reduction in working hours compared to employees. Self-employed individuals are also about six percentage points more likely to work from home.

**Table 1:** Restricted and unrestricted models for differences in the likelihood that income or working hours decreased or individuals are working from home between employees and self-employed respondents

	(1)	(2)	(3)	(4)	(5)	(6)
	Income	Income	Working	Working	Remote	Remote
			hours	hours	work	work
Self-employed	0.418***	0.421***	0.301***	0.302***	0.061**	0.021
	(0.029)	(0.031)	(0.029)	(0.031)	(0.030)	(0.032)
Demographics						
Gender: female		0.019		0.022		− 0.013
		(0.013)		(0.016)		(0.017)
Age		0.006		− 0.003		− 0.005
		(0.005)		(0.005)		(0.005)
Age squared		0.000		0.000		0.000
		(0.000)		(0.000)		(0.000)
Migration background		0.040**		0.040**		− 0.026
		(0.016)		(0.019)		(0.019)
Big 5						
Extraversion		0.000		0.008		− 0.001
		(0.006)		(0.007)		(0.008)
Conscientiousness		− 0.010		− 0.018**		0.001
		(0.007)		(0.008)		(0.008)
Openness to experience		0.010		0.006		0.025***
		(0.006)		(0.007)		(0.008)
Neuroticism		− 0.004		0.001		− 0.008
		(0.006)		(0.007)		(0.007)
Agreeableness		0.004		− 0.004		0.002
		(0.006)		(0.007)		(0.008)
Household context						
HH size		0.006		0.011		− 0.008
		(0.007)		(0.008)		(0.009)
Married		0.021		0.016		− 0.021
		(0.015)		(0.017)		(0.018)
School child or younger		0.007		− 0.004		0.049**
		(0.018)		(0.021)		(0.022)
Log of HH net income		− 0.039**		− 0.034*		0.098***
		(0.016)		(0.018)		(0.020)
Education (ref. low)						
Intermediate education		0.031		0.023		0.073***
		(0.019)		(0.022)		(0.020)
High education		0.011		− 0.005		0.293***
		(0.021)		(0.024)		(0.024)
Unemployment experience		0.000		0.005*		− 0.005**
		(0.003)		(0.003)		(0.002)
Mean of outcome	0.169	0.169	0.222	0.222	0.395	0.395
Observations	3531	3531	3518	3518	3533	3533
*R*^2^	0.11	0.23	0.05	0.13	0.03	0.31

The table displays models with and without controls for differences between self-employed and employees. All models include state and week fixed effects. Columns (1), (3), and (5) display results for the models without controls. Columns (2), (4), and (6) display results for the models with controls. The unrestricted models also include NACE 2 fixed effects. Standard errors are robust and in parentheses. *p < 0.10, **p < 0.05, ***p < 0.01

The comparison of odd-numbered with even-numbered columns of Table 1 reveals that individual-level and household-level characteristics explain very little of the differences between self-employed individuals and employees with respect to the probability of income losses and hours reductions. The coefficient on the indicator for self-employment remains almost unchanged when adding controls (compare column (1) to column (2) and column (3) to column (4), respectively). Having a migration background appears to significantly increase the probability of suffering income losses and hours reductions, while a higher household income has the opposite effect. That is consistent with the finding of Fairlie (2020), who also finds a racial gap in how the self-employed are hit by the COVID-19 pandemic. By contrast, the probability of working from home seems to be explained by the added controls: Individuals from more affluent households are more likely to be working from home during the pandemic, likely a result of selection into jobs that are more easily done from home (e.g., office jobs; see Alipour et al. 2020). Similarly, better-educated individuals are significantly more likely to work from home, so are parents.

To pin down the relevance of industry fixed effects, Table 9 in Appendix 1 displays the *R*-squared alongside the coefficients on the self-employment indicator for the unrestricted models in Table 1, both with and without the inclusion of industry fixed effects. The *R*-squared increases substantially once industry effects are accounted for, implying that industry-variation contributes significantly to explaining the respective outcomes.^10^ However, differential selection into industries adds rather little to describing the overall differences between employees and the self-employed, as evidenced by the marginal changes in the self-employment gap once industry fixed effects are accounted for.^11^

In summary, it seems that the differential impact of the COVID-19 pandemic between employees and the self-employed with respect to income and working hours is neither primarily driven by differences in individual- and household-level characteristics nor by selection into different industries, but by differences in the association of these factors with the respective outcomes. The pandemic shock hit the self-employed uniformly harder. This seems plausible as employees are often shielded from job and income losses by employment contracts and job protection legislation, while such mechanisms do not exist for the self-employed. By contrast, individual- and household-level characteristics can nearly fully account for differences in the likelihood of working from home between self-employed and employed individuals.

Thus far, we focus our analysis on the population of (self-)employed individuals in 2020. However, employees may have lost their job over the course of the pandemic and self-employed individuals may have terminated their business. To account for this, we look at the working population of 2019 and investigate whether individuals who were self-employed in 2019 differ from those who were employees with respect to the probability of changes in income, changes in working hours, and job loss. The latter is defined as the proportion of individuals who transitioned into non-employment between 2019 and 2020 and who respond that this transition was due to the COVID-19 pandemic. The results are shown in Table 11 in Appendix 1. Overall, 1.7% of those working in 2019 are non-employed in 2020 because of the pandemic. Importantly, self-employed individuals are 1.2 percentage points more likely to have terminated their business than employees are to have lost their job, although this difference is not statistically significant. Note as well that the reported results for income and working hours changes slightly differ from those in Table 1. This is explained by the focus on the employment status of 2019, rather than 2020 in Table 11 in Appendix 1. Differences result from two sources: First, employees surveyed in 2019 may have become self-employed between the times of the interview in 2019 and 2020, and vice versa. Second, individuals who were not in employment at the time of the interview in 2019 may have founded a business prior to the time of the interview in 2020. However, the differences in the reported results between Table 1 and Table 11 in Appendix 1 are minor.

### Gender differences among the self-employed

As discussed in Section 3.3, we observe considerable gender differences in the probability of income declines among the self-employed. Section 4.1 further reveals that self-employed individuals are, in general, much more likely to suffer income losses than employees. Turning to our core analysis, we investigate how self-employed as well as employed women are affected by the COVID-19 pandemic in comparison to their male counterparts. We apply the Gelbach (2016) decomposition to further analyze the gender differences with respect to the likelihood of a decline in income due to the COVID-19 pandemic. This decomposition reveals the individual contributions of covariates to the change in the gender gap, thus assigning each covariate-bundle a proportion of the overall contribution. Importantly, it is not path dependent, as this decomposition is, unlike sequential covariate addition, invariant to the sequence in which we would usually insert the covariates to gauge the stability of the coefficient of interest. In our analysis, the Gelbach decomposition answers the question of how much of the change in the gender gap can be attributed to different variables in the set of controls as we move from the base specification, the restricted model, to the full specification that includes all controls, the unrestricted model (for more details on the methodology, see Appendix 2).

In our sample of self-employed individuals, we observe a gender gap of 17.4 percentage points in the likelihood of experiencing an income loss in our restricted model. This can be inferred from column (1) in Table 2.^12^ Relative to self-employed men, self-employed women are about one-third more likely to experience an income loss because of the COVID-19 pandemic. As discussed in Section 3.3 and confirmed in Table 13 in Appendix 1, there is no comparable gender gap among employees. In our unrestricted model in column (2) of Table 2, the gender gap decreases to 8.1 percentage points and is statistically indistinguishable from zero. This outcome implies that our controls can explain about 9.3 percentage points, or 53.4%, of the initial gender gap.^13^

**Table 2:** Restricted and unrestricted models for the likelihood that income or working hours decreased or individuals are working from home among self-employed individuals

	(1)	(2)	(3)	(4)	(5)	(6)
	Income	Income	Working	Working	Remote	Remote
			hours	hours	work	work
Gender: female	0.174***	0.081	0.068	− 0.051	− 0.017	− 0.040
	(0.058)	(0.073)	(0.060)	(0.073)	(0.057)	(0.069)
Demographics						
Age		0.027		0.007		− 0.042**
		(0.019)		(0.020)		(0.021)
Age squared		− 0.000*		0.000		0.000*
		(0.000)		(0.000)		(0.000)
Migration background		0.064		0.120		− 0.117
		(0.110)		(0.099)		(0.085)
Big 5						
Extraversion		0.011		0.067*		0.046
		(0.040)		(0.037)		(0.037)
Conscientiousness		− 0.031		− 0.058		0.033
		(0.039)		(0.038)		(0.037)
Openness to experience		0.066*		0.051		0.058*
		(0.038)		(0.036)		(0.034)
Neuroticism		− 0.031		− 0.003		− 0.013
		(0.036)		(0.039)		(0.035)
Agreeableness		− 0.040		− 0.067*		− 0.032
		(0.035)		(0.034)		(0.033)
Household context						
HH size		− 0.061		− 0.076**		0.092***
		(0.039)		(0.036)		(0.033)
Married		0.037		− 0.010		0.026
		(0.073)		(0.078)		(0.071)
School child or younger		0.045		0.211**		− 0.018
		(0.103)		(0.094)		(0.101)
Log of HH net income		− 0.026		0.100*		− 0.146***
		(0.058)		(0.058)		(0.052)
Education (ref. low)						
Intermediate education		− 0.102		0.074		− 0.108
		(0.125)		(0.114)		(0.112)
High education		− 0.149		− 0.026		0.057
		(0.132)		(0.120)		(0.119)
Unemployment experience		− 0.026**		0.001		− 0.013
		(0.012)		(0.010)		(0.011)
Mean of outcome	0.552	0.552	0.495	0.495	0.457	0.457
Observations	310	310	309	309	311	311
*R*^2^	0.13	0.41	0.09	0.40	0.16	0.47

The table displays restricted and unrestricted models underlying the Gelbach decomposition. All models include state and week fixed effects. Columns (1), (3), and (5) display results for the restricted models. Columns (2), (4), and (6) display results for the unrestricted models. The unrestricted models also include NACE 2 fixed effects. Standard errors are robust and in parentheses. *p < 0.10, **p < 0.05, ***p < 0.01

**Fig. 6:**
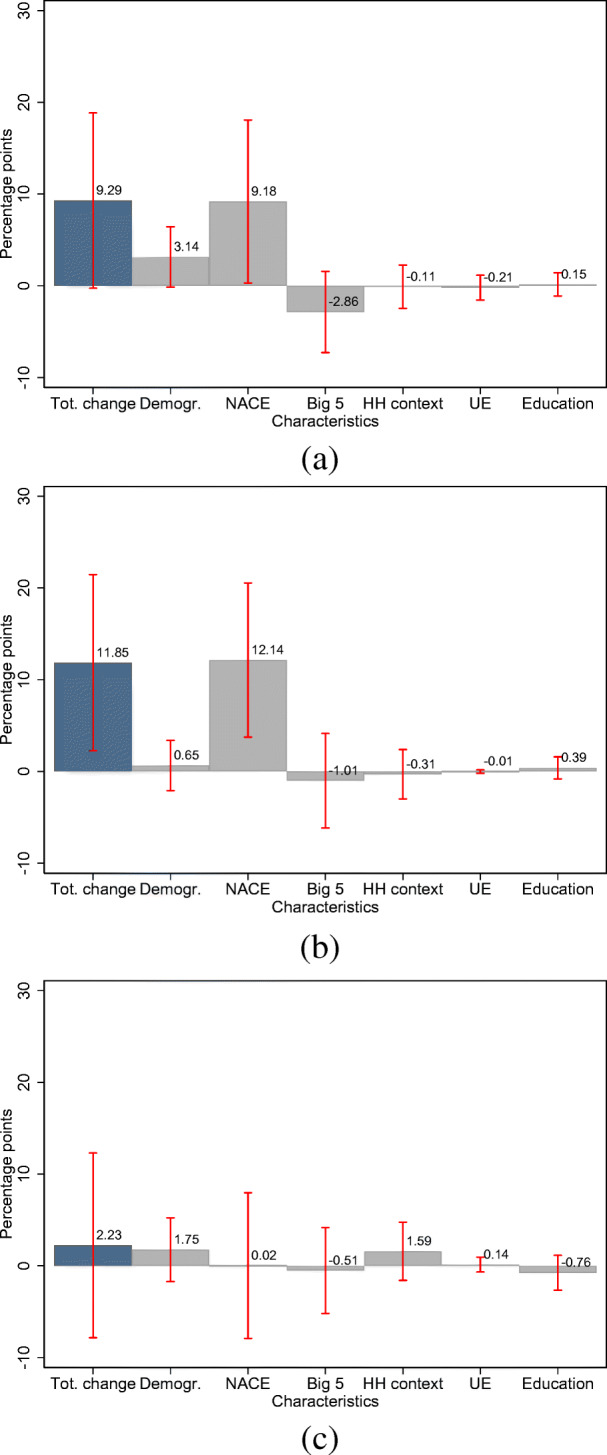
Gelbach decomposition of the gender gap in labor market outcomes among self-employed respondents. Note: a-c display the Gelbach decomposition of the gender gap in the likelihood of an income reduction, a reduction in working time, and working from home among self-employed respondents. Red bars indicate 95% confidence intervals based on robust standard errors. **a** Likelihood of income decline. **b** Likelihood of decline in working time. **c** Likelihood of remote work

The largest share of the gender gap in income losses is explained by the fact that women are over-represented in industries in which individuals are more likely to experience income losses. This is seen in Fig. 6a, which displays the results of the Gelbach decomposition: 9.2 percentage points, or 98.8% of the total change, can be explained by NACE fixed effects.^14^ Demographic characteristics, particularly age, explain as much as 33.8% of the total change in the gender gap between the unrestricted and restricted models. Other groups of characteristics add nearly nothing to the total change in the gender gap.

**Fig. 7:**
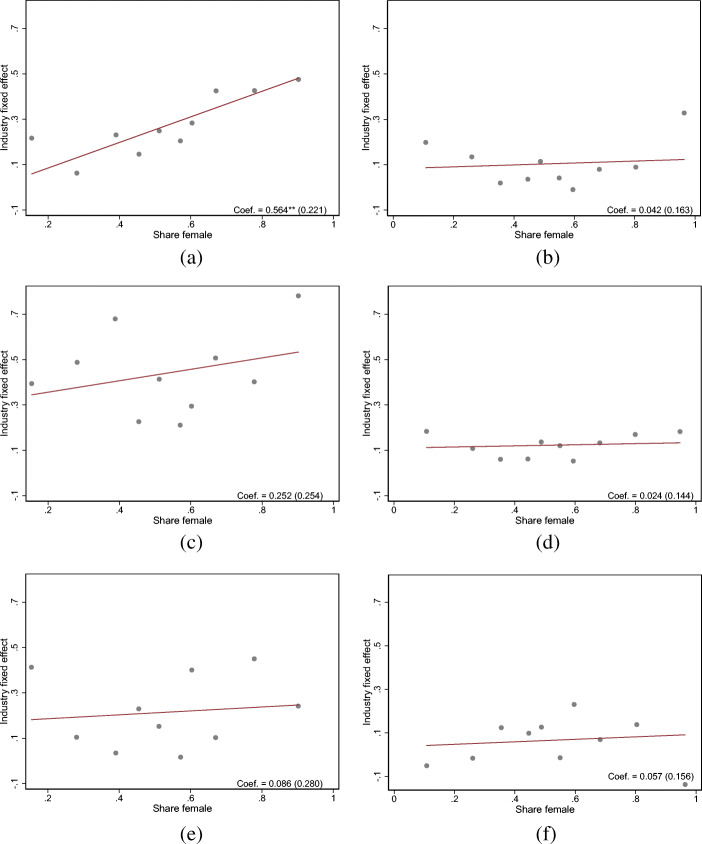
The association between industry specific fixed effects for the probability of an income or working time decrease as well as for the probability of working from home and the share of women in the respective industry. Note: **a**–**f** display the association between industry specific fixed effects and the share of women in the respective industry for the working population in 2020. The fixed effects stem from a regression of our three outcomes on industry indicators, respectively. The share of women corresponds to the share of women in the respective industry in our working sample. Both figures correspond to a binned scatterplot. The regression coefficients stem from an OLS regression of the industry fixed effects on the share of women in the respective industries. Robust standard errors are in parentheses. *p < 0.10, **p < 0.05, ***p < 0.01. **a** Income decline for self-employed individuals. **b** Income decline for employees. **c** Working time decline for self-employed individuals. **d** Working time decline for employees. **e** Remote work for self-employed individuals. **f** Remote work for employees

Thus, the industry-specific likelihood of an income loss is positively associated with the share of women in the respective industry. In Fig. 7, we display binned scatter plots for the association between the respective industry-specific fixed effects in the likelihood of an income loss and the share of women for self-employed individuals and employees, respectively.^15^ We observe a positive association between the industry fixed effects and the share of women in the respective industries. The OLS coefficient for the underlying relationship implies that a 10 percentage point higher share of women in a given industry is associated with an increase in the likelihood of experiencing an income loss of about 5.6 percentage points.

Moreover, the results in columns (3) and (5) of Table 2 do not support the notion of a gender gap in the likelihood of a decline in working hours and working from home.^16^,^17^ However, the change in the OLS coefficient for the indicator for being female between the restricted and unrestricted model and Fig. 6b suggests an economically significant change in the likelihood of a decline in working hours of about 11.9 percentage points. This is more

than fully accounted for by the fact that, again, women are disproportionately represented in those industries, which are hit the hardest by the COVID-19 pandemic. In addition, Fig. 7c suggests a positive association between the share of women across industries and the likelihood of experiencing a decline in working hours in these industries. This constitutes evidence that the industry affiliation moderates the relationship between the likelihood of a decline in working hours and the gender of self-employed respondents, while there is no evidence for such a relationship for the probability of working from home. We also do not find support for such a relationship among employees.

In Table 12 in Appendix 1, we display the five industries with the highest and lowest shares of women, respectively. The industries with the highest share of women include, for example, the hospitality sector and personal services — industries that were hit particularly hard by the COVID-19 crisis.^18^ For each of these industries we also show the associated industry fixed effect from a regression of the likelihood of an income loss on state and week indicators as well as industry indicators. The average share of women for these industries in our sample is 82.57% and the average estimate of the fixed effects is 0.41.^19^ Conversely, the average share of women in the five industries with the lowest shares of women in our sample is 25.68% and the average fixed effect for these industries is 0.19.^20^ Thus, the contribution of industry fixed effects to the likelihood of suffering income losses due to the COVID-19 pandemic is largest in industries where women are overrepresented.

## Potential mechanisms

In this section, we investigate potential mechanisms driving our results. Note that the gendered industry effects presented in Section 4.2 encompass a variety of factors: Not only do they suggest the existence of direct effects of the pandemic that impact industries to varying degrees, i.e., through government-imposed restrictions, but also the importance of other NPIs, such as the closure of schools or day-care centers, and the indirect effects these have on income or hours worked, i.e., through changes in the intra-household allocation of time. Therefore, the overall contribution of the industry fixed effects is the product of the strength of the selection into industries as well as the association of the respective industry with the respective outcome.

In the following, we further characterize these relationships. We investigate to what extent direct regulations, or shortages in supply or demand, drive the disproportionate impact of the COVID-19 pandemic on self-employed women. We then test whether gendered specialization in home production might have contributed to the differential impact of the COVID-19 pandemic among self-employed women and men.

### Business-related distortions due to the COVID-19 pandemic

In the SOEP-CoV questionnaire, self-employed respondents were asked whether they have been affected by several events in the wake of the COVID-19 pandemic and associated NPIs. Of these, we focus on events that might have detrimental effects on the self-employed respondents’ income or working time. These are “being affected by regulations, e.g. opening hours” (restrictions), “suppliers are not able to deliver parts or products to perform business” (supply), and “customers are cancelling services or orders” (demand). We apply the Gelbach decomposition to decompose the gender gap in the likelihood that the self-employed respondents report to have been affected by these events. Table 3 displays the restricted and unrestricted model for these three events.^21^

**Table 3:** Restricted and unrestricted models for the likelihood that a business was affected by the respective event

	(1)	(2)	(3)	(4)	(5)	(6)
	Restrictions	Restrictions	Supply	Supply	Demand	Demand
Gender: female	0.202***	0.051	− 0.027	− 0.057	0.052	− 0.007
	(0.058)	(0.068)	(0.041)	(0.048)	(0.059)	(0.073)
Demographics						
Age		− 0.005		0.028**		0.022
		(0.019)		(0.013)		(0.019)
Age squared		0.000		− 0.000**		− 0.000*
		(0.000)		(0.000)		(0.000)
Migration background		0.092		0.014		0.032
		(0.090)		(0.075)		(0.097)
Big 5						
Extraversion		0.039		− 0.004		0.039
		(0.037)		(0.029)		(0.039)
Conscientiousness		− 0.025		0.021		− 0.046
		(0.036)		(0.024)		(0.039)
Openness		− 0.030		− 0.009		0.055
		(0.037)		(0.027)		(0.038)
Neuroticism		0.064*		− 0.001		0.001
		(0.035)		(0.024)		(0.039)
Agreeableness		0.037		− 0.038		− 0.017
		(0.035)		(0.026)		(0.037)
Household context						
HH size		− 0.001		0.024		− 0.035
		(0.032)		(0.027)		(0.040)
Married		− 0.019		− 0.058		− 0.041
		(0.073)		(0.056)		(0.079)
School child or younger		− 0.091		− 0.099		− 0.038
		(0.096)		(0.078)		(0.108)
Log of HH net income		− 0.057		0.015		0.018
		(0.057)		(0.044)		(0.060)
Education (ref. low)						
Intermediate education		− 0.110		− 0.147		− 0.112
		(0.105)		(0.098)		(0.116)
High education		− 0.054		− 0.132		− 0.100
		(0.108)		(0.103)		(0.120)
Unemployment experience		− 0.016		− 0.011**		− 0.021**
		(0.011)		(0.005)		(0.009)
Mean of outcome	0.457	0.457	0.122	0.122	0.434	0.434
Observations	311	311	311	311	311	311
*R*^2^	0.13	0.46	0.05	0.31	0.09	0.38

The table displays restricted and unrestricted models underlying the Gelbach decomposition for business events. All models include state and week fixed effects. Columns (1), (3), and (5) display results for the restricted models. Columns (2), (4), and (6) display results for the unrestricted models. The unrestricted models also include NACE 2 fixed effects. Standard errors are robust and in parentheses. *p < 0.10, **p < 0.05, ***p < 0.01

**Fig. 8:**
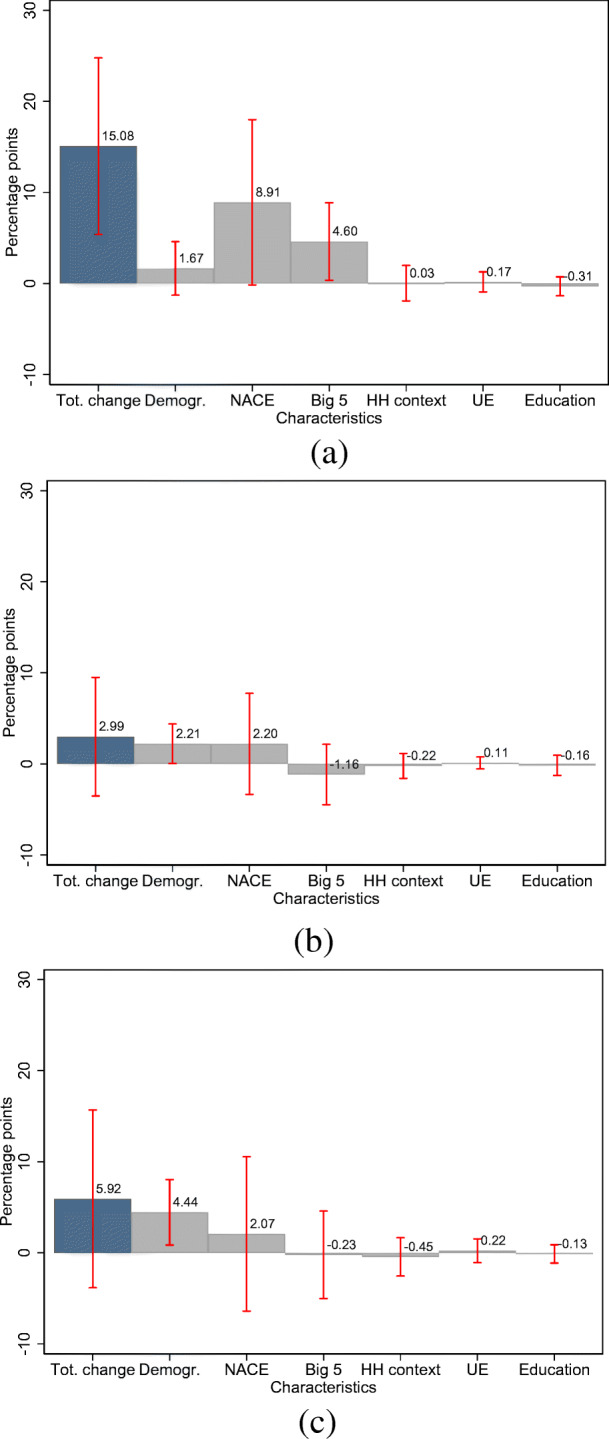
Gelbach decomposition of the gender gap in business-related events. Note: **a**–**c** display the Gelbach decomposition of the gender gap in the likelihood of various business-related events. Red bars indicate 95% confidence intervals and are based on robust standard errors. **a** Rules or restrictions. **b** Supply of intermediate products **c** Demand shortage

We find that self-employed women are 20.2 percentage points more likely than their male counterparts to state that they are affected by rules or restrictions. We do not find such differences for the supply of intermediate goods or for demand shortages. In Fig. 8, we show detailed Gelbach decompositions of the gender gap for business-related events. The Gelbach decomposition in Fig, 8, along with the results in Table 3, provide evidence that it is, once again, the disproportionate representation of women in industries most affected by the pandemic that explains the differential effects.^22^ Our full set of covariates explains about 15 percentage points of this gender gap, with about 9 percentage points thereof attributable to industry fixed effects. While the total change of the gender gap between the restricted and unrestricted model is significant at the five percent level of significance, the contribution of industry fixed effects is significant at the ten percent level of significance.

**Fig. 9:**
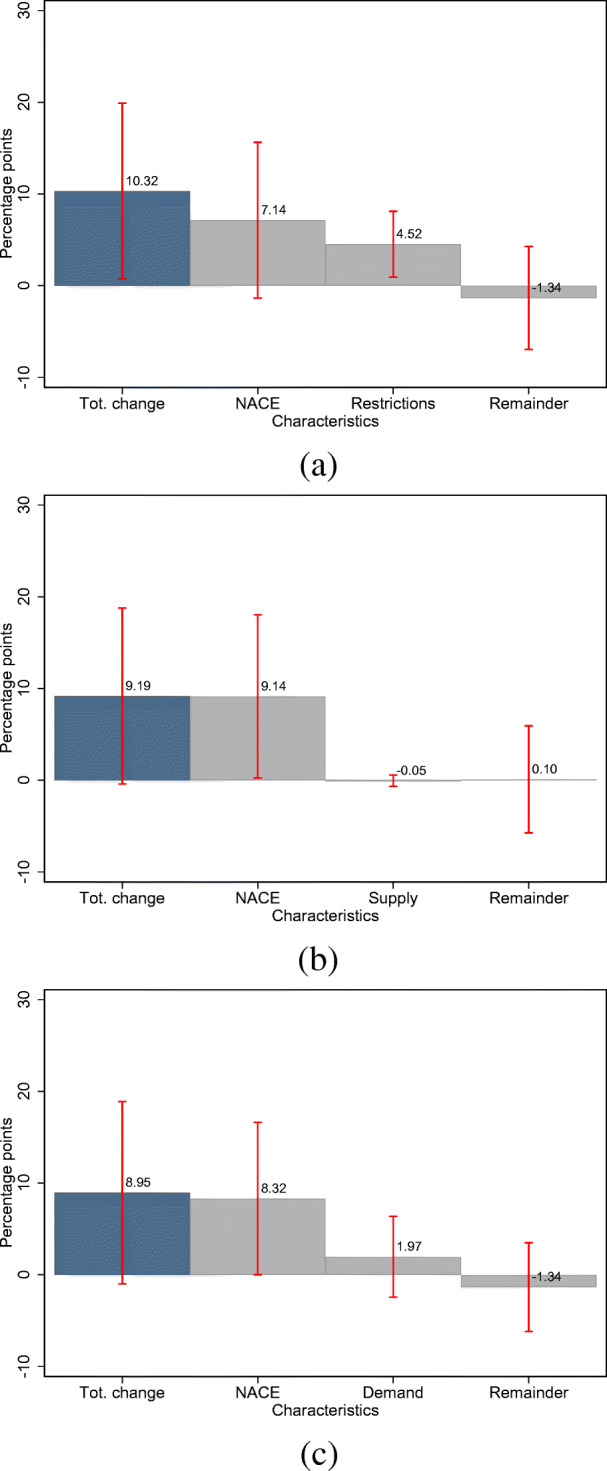
Contribution of the business-related events to the gender gap in the likelihood of an income decline. Note: **a**–**c** display the importance of various business-related events for the gender gap in the likelihood of an income decline. We summarize the residual characteristics in the category “Remainder.” Red bars indicate 95% confidence intervals and are based on robust standard errors. **a** Rules or restrictions. **b** Supply of intermediate products. **c** Demand shortage

Moreover, we find that government-imposed restrictions contribute significantly to the gender gap in the likelihood of an income decline. This is shown in Fig. 9, where we include indicators for the three business-related events in the wake of the COVID-19 pandemic in the Gelbach decomposition of the gender gap in income losses.^23^ Among the three business-related events considered, being affected by rules and restrictions due to the COVID-19 pandemic is the only relevant contributor to the gender gap in income loss. As depicted in Fig. 9, rules and restrictions account for 4.5 percentage points of the total change of 10.3 percentage points.^24^ At the same time, the contribution of industry fixed effects is considerably attenuated from 9.2 to 7.1 and is significant at the 10% level of significance, suggesting that government-imposed restrictions disproportionately affect industries in which women are over-represented and that those restrictions contribute to positively to the likelihood of an income decline.

### Household income and household chores

As noted previously, direct regulations of businesses are not the only government interventions that can potentially affect labor market outcomes of self-employed individuals. Other NPIs include the closure of schools and child-care facilities, which may also contribute to the observed gender gap. Assume that households maximize income subject to a time constraint. Further, assume decreasing returns and comparative advantages in household and market production, respectively. Under these conditions, both spouses would participate in the labor force in normal times. However, their respective contributions to the household income would be determined by their relative productivity in home and market production (e.g., Weiss 1993; Bertrand et al. 2015). In this class of models, the partner who is relatively more productive at home production tends to spend more time with household chores or childcare. At the same time, their spouse spends more time in market production, where they are hypothesized to be relatively more productive, and thus earn a higher income.^25^

Given these assumptions, households need to re-optimize if, for instance, child-care facilities close. Under these circumstances, it is likely that the partner with the higher relative productivity in home production reduces time in market production while the other partner increases hours worked, *ceteris paribus*. One implication of this simplified model is that, if women tend to be the partner who is relatively more productive in home production, we would observe a gender gap in income and time decreases as a consequence of NPIs reducing the share of home production that can be outsourced, i.e., the closure of childcare facilities.^26^ So far, we have accounted for this by controlling for the presence of children and household size.

We now test this prediction by including an individual’s earnings, relative to the overall earnings of the household, in our models. The concept is focal in the literature on gender norms (e.g., Bertrand et al. 2015; Foster and Stratton 2021). For each respondent, we know the partner from 2018. Thus, we are able to link the partners’ earnings from 2019 to each respondent. Then, we calculate the relative earnings of each individual within each of these household pairs. Note that not every individual in our data has a partner. In such cases, the relative earnings for this observation is 100% or 0%. We account for these single households via the inclusion of an indicator for having a partner in 2018. If an individual did not work in 2019, we impute zero earnings. We then include relative earnings in the Gelbach decomposition. If the conjecture above is true, we would expect that women are more likely to have lower relative earnings and relative earnings would be negatively associated with the incidence of a decrease in working time, income, or the likelihood of working from home.

**Fig. 10:**
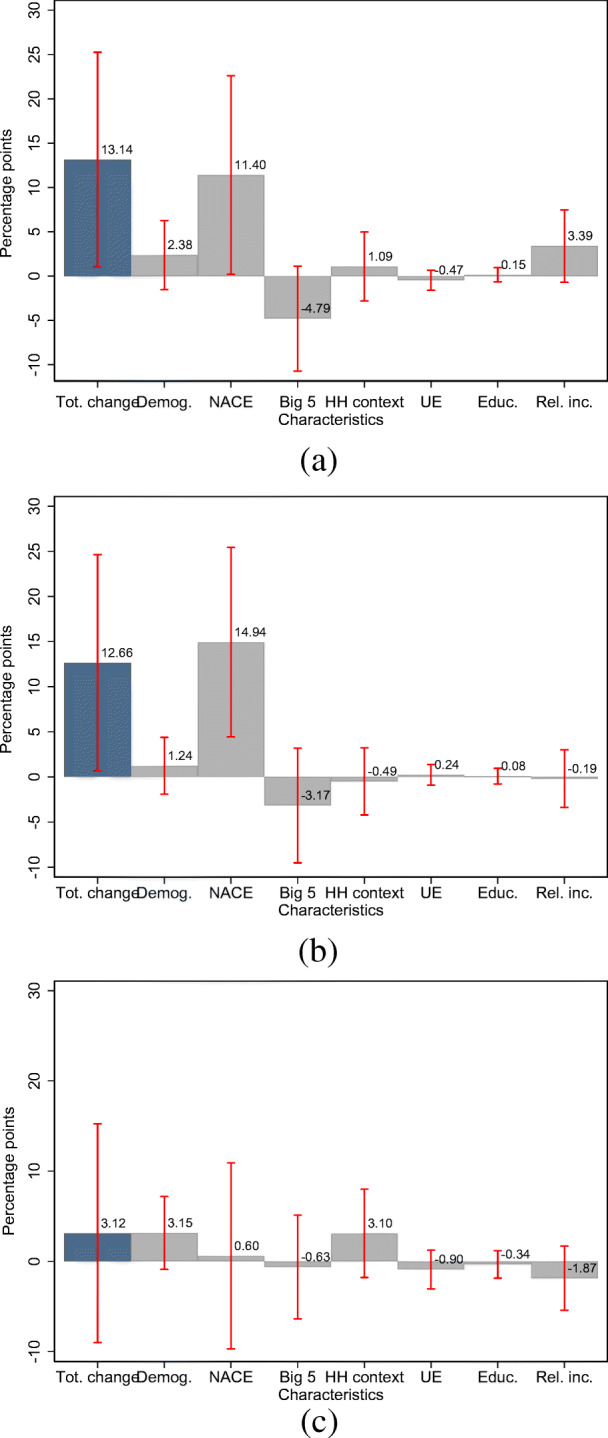
Gelbach decomposition of the gender gap in labor market outcomes among self-employed respondents, testing for specialization in the household context. Note: **a**–**c** display the Gelbach decomposition of the gender gap in the likelihood of an income reduction, a reduction in working time, and working from home among self-employed respondents. Red bars indicate 95% confidence intervals based on robust standard errors. **a** Reduction of income. **b** Reduction in weekly working hours. **c** Remote work

With respect to the likelihood of income reductions we find some evidence for the first part of the conjecture. That is, the results indicate that households optimize and exploit comparative advantages. Table 4 displays the restricted and unrestricted model for our outcome variables. In addition to the standard set of controls, we now include the individual’s share of household earnings in 2019. In addition, all models include an indicator for the presence of a partner. For the likelihood of an income decline due to the COVID-19 pandemic, the earnings share of the individual is significant at the ten percent level of significance. The point estimate suggests that a ten percent increase in the individual’s earnings share is associated with a 2.6 percentage points reduction in the likelihood of an income reduction. Similarly, the Gelbach decomposition in Fig. 10a suggests that women account for a smaller share of the total household earnings, on average, and that the share of household earnings is negatively associated with the likelihood of an income reduction due to the COVID-19 pandemic. This relationship accounts for 25.8% of the total change in the gender gap. However, the estimate is not very precisely estimated, meaning we cannot reject the hypothesis that this contribution is not different from zero (*p* = 0.104). However, it is worth emphasizing that the gender gap almost completely vanishes as soon as we account for relative earnings (compare column (2) of Table 2 to column (2) of Table 4).

**Table 4:** Restricted and unrestricted models for differences in likelihood that income or working hours decreased, accounting for relative income differences

	(1)	(2)	(3)	(4)	(5)	(6)
	Income	Income	Working hours	Working hours	Remote work	Remote work
Gender: female	0.154**	0.022	0.066	− 0.054	0.002	− 0.029
	(0.067)	(0.086)	(0.070)	(0.088)	(0.066)	(0.077)
Demographics						
Age		0.056*		0.016		− 0.031
		(0.032)		(0.033)		(0.034)
Age squared		− 0.001*		0.000		0.000
		(0.000)		(0.000)		(0.000)
Migration background		0.018		0.025		− 0.207*
		(0.131)		(0.114)		(0.113)
Big 5						
Extraversion		0.044		0.054		0.052
		(0.047)		(0.045)		(0.044)
Conscientiousness		− 0.040		− 0.016		− 0.016
		(0.046)		(0.045)		(0.045)
Openness to experience		0.055		0.035		0.048
		(0.048)		(0.046)		(0.041)
Neuroticism		− 0.062		− 0.042		− 0.015
		(0.042)		(0.044)		(0.040)
Agreeableness		− 0.087**		− 0.073*		− 0.023
		(0.043)		(0.043)		(0.041)
Household context						
HH size		− 0.072		− 0.065		0.100***
		(0.050)		(0.043)		(0.036)
Married		0.072		− 0.012		0.028
		(0.124)		(0.151)		(0.117)
School child or younger		0.056		0.247**		0.078
		(0.124)		(0.110)		(0.124)
Log of HH net income		− 0.064		− 0.127**		− 0.127**
		(0.069)		(0.066)		(0.064)
Education (ref. low)						
Intermediate education		0.019		0.090		− 0.049
		(0.146)		(0.137)		(0.137)
High education		− 0.033		0.064		0.065
		(0.161)		(0.142)		(0.149)
Unemployment experience		− 0.025		0.013		− 0.048***
		(0.020)		(0.021)		(0.017)
Income share		− 0.260*		− 0.002		0.143
		(0.135)		(0.156)		(0.136)
Mean of outcome	0.561	0.561	0.496	0.496	0.496	0.496
Observations	239	239	238	238	238	238
*R*^2^	0.17	0.50	0.13	0.48	0.13	0.48

The table displays restricted and unrestricted models underlying the Gelbach decomposition. All models include state and week fixed effects as well as indicators for having a partner. Columns (1), (3), and (5) display results for the restricted models. Columns (2), (4), and (6) display results for the unrestricted models. The unrestricted models also include NACE 2 fixed effects. Standard errors are robust and in parentheses. *p < 0.10, **p < 0.05, ***p < 0.01

With respect to the likelihood of a reduction in working hours or the incidence of working from home, we find no evidence for a significant association with the individuals’ earnings share within the household. The Gelbach decompositions in Figs. 10b and c likewise do not provide an indication that the relative income position contributes to explaining the gender gap. One interpretation of these findings is that a negative association would appear only for outcomes that translate directly into material well-being. For working time, this is not clear a priori. For self-employed individuals, there are various possible circumstances where working time reductions do not necessarily translate into reduced earnings. With respect to the incidence of working from home, other factors are likely more relevant, i.e., the extent to which the job of the self-employed individual or their partner can be performed remotely.^27^

## Conclusion

We analyze how the economic shock related to SARS-CoV-2 affects the self-employed in comparison to employees, and focus in particular on the female working population. We show that the 4.2 million self-employed men and women are 42 percentage points more likely to experience an income loss than employees and that they have a 30 percentage points higher chance of experiencing a decrease in working hours. This differential impact on the two employment forms cannot be explained by differences in individual-level characteristics or selection into different industries. The self-employed are more likely to suffer income losses and reductions in working hours throughout.

Unlike for self-employed workers, employees’ wages and working hours in Germany are more rigid than in comparable countries. In addition, to prevent mass layoffs, the German government expanded “*Kurzarbeit*,” its well-established short-time work scheme that allows for temporary reductions in the wages and hours of employees. Indeed, the fraction of employees who experience income losses is proportional to the fraction of employees in short-time work schemes (Kritikos et al. 2020). Thus, it appears that the labor market impact of the COVID-19 pandemic was mitigated by “*Kurzarbeit*”.

Among the self-employed, we find that women are about one-third more likely to face income losses due to the COVID-19 pandemic than men. We do not find a comparable gender gap among employees, which is likely a result of labor market rigidities. Our results reveal that the largest share of gender differences among the self-employed is attributable to the fact that self-employed women work disproportionately often in industries that are more severely affected by the COVID-19 pandemic than men. This is supported by the observable gender gap in the extent to which self-employed individuals were affected by government-imposed restrictions, such as the regulation of opening hours. We provide evidence that this directly translates into gender differences in income losses. Moreover, we find suggestive evidence that gendered household production also contributes to the gender gap in income losses. Still, this is of second order compared to the contribution of industry effects.

Our study has important policy implications that may be applicable to policy responses to the further development of the current pandemic or for future pandemics (Petrovan et al. 2020). We show that the self-employed, in particular women, are hit significantly harder by this systemic shock than other parts of the working population, which is, in part, a direct consequence of policy measures enacted to contain the spread of the virus. This outcome should also be seen in the context of the slowly increasing willingness of women to enter self-employment. If self-employed women feel less supported by policy measures during such a systemic shock than female employees, society risks that they will start turning away from this employment form. Thus, the gender gap in self-employment may widen again. This could negatively affect growth, notably in parts of the economy that depend strongly on female self-employment. The design of policy measures intending to mitigate negative economic shocks in the ongoing or in comparable future crisis situations, should, therefore, account for this variation in economic hardship. Given our finding that government-imposed restrictions are a factor through which this unequal impact of the pandemic emerges, targeted policies that restore gender equity seem particularly relevant. Similarly, given our finding that the self-employed are disproportionately affected by the COVID-19 pandemic, policy makers may consider different measures aimed at supporting them. However, every such policy measure involves the risk of moral hazard. That is, it provides incentives for self-employed individuals to engage in risky behavior in a way it would not occur in the absence of support schemes. On the other hand, the detrimental effect of the COVID-19 pandemic on the self-employed is not the result of individual decision-making, rather it is a systematic and unexpected shock, and in part a direct consequence of government regulation. More generally, any support scheme for the self-employed may create both negative and positive externalities, which are to be weighted against each other. For instance, self-employment and entrepreneurship are shown to have a positive effect on growth (Stoica et al. 2020). As such, support schemes which successfully retain the propensity to remain self-employed through the crisis have the potential to facilitate recovery after the COVID-19 pandemic.

## Appendix 1. Additional tables

**Table 5:** Variable descriptions

(1)	(2)	(3)
Variable	Description	Year of origin
Income (gross) decrease	Indicator reflecting decrease of monthly gross income due to the COVID-19 pandemic.	2020
Working hour decrease	Indicator reflecting decrease of weekly working hours due to the COVID-19 pandemic.	2020
Income loss	Exact amount of lost income due to the COVID-19 pandemic.	2020
Number of working hour decrease	Number of weekly working hours decreased due to the COVID-19 pandemic.	2020
Remote work	Indicator reflecting working from home due to the COVID-19 pandemic.	2020
Age	Difference between survey year and birth year.	Pre-2020
Female	Indicator for being female.	Pre-2020
Migration background	Indicator for having a direct or indirect migration background.	Pre-2020
Openness to experience	Second factor of a principal component analysis of the items of the BIG 5-inventory.	2019
Conscientiousness	Third factor of a principal component analysis of the items of the BIG 5-inventory.	2019
Extraversion	First factor of a principal component analysis of the items of the BIG 5-inventory.	2019
Agreeableness	Fifth factor of a principal component analysis of the items of the BIG 5-inventory.	2019
Neuroticism	Fourth factor of a principal component analysis of the items of the BIG 5-inventory.	2019
Household size	Number of household members.	2019
Household net income	Monthly household net income in 2015 Euros. If information is missing, we imputed the information by plugging in the mean for each education x child presence x self-employment status-cell.	2019
Married	Indicator for being married.	2019
School child or younger	Indicator reflecting the presence of a child of school age or younger.	2020
Basic school leaving degree	Indicator for categories 0 “in school” to 1c “basic vocational education” according to the Comparative Analysis of Social Mobility in Industrial Nations (CASMIN)-scale.	Last available information in seven years pre 2020
Intermediate school leaving degree	Indicator for categories 2b “intermediate general qualification” to 2c_voc “vocational maturity certificate” according to the CASMIN-scale.	Last available information in seven years pre 2020
Tertiary school leaving degree	Indicator for categories 3a “lower tertiary education” or 3b “higher tertiary education” according to the CASMIN-scale.	Last available information in seven years pre 2020
Unemployment experience	Generated unemployment experience from “pgen.dta” of the SOEP v.35.	2018
NACE 2 code	Two-digit NACE Industry – Sector. Missing values, e.g., due to unemployment in 2019, are coded as a separate category.	2019
Subject to regulation	Indicator reflecting whether the self-employed individual’s business was subject to regulations to contain COVID-19, e.g., regulation of opening hours.	2020
Supply problems	Indicator reflecting whether the self-employed individual’s business suffered from shortages of intermediate goods.	2020
Demand problems	Indicator reflecting whether the self-employed individual’s business suffered from cancellation of their services and goods, i.e., demand shortage.	2020

The table provides information on variables and their year of origin

**Table 6:** Summary statistics

	(1)	(2)		(3)	(4)	(5)
	Self-employed	Individuals		Employees	Individuals	*P*-value of (1)–(3)
Income (gross) decrease	0.552	310		0.132	3221	0.000
Working hour decrease	0.495	309		0.196	3209	0.000
Remote work	0.457	311		0.390	3222	0.021
Demographics						
Age	53.791	311		47.034	3222	0.000
	(11.154)			(10.533)		
Female	0.498	311		0.611	3222	0.000
Migration background	0.164	311		0.205	3222	0.086
Personality traits						
Openness to experience	0.317	311		− 0.032	3222	0.000
	(1.010)			(0.975)		
Conscientiousness	0.099	311		0.076	3222	0.664
	(0.928)			(0.919)		
Extraversion	0.092	311		0.015	3222	0.196
	(0.967)			(1.019)		
Agreeableness	− 0.005	311		− 0.088	3222	0.159
	(1.009)			(0.989)		
Neuroticism	− 0.127	311		− 0.051	3222	0.188
	(0.954)			(0.973)		
Household context						
Household size	2.617	311		2.815	3222	0.017
	(1.427)			(1.386)		
Household net income (€)	4619.53	311		3826.88	3222	0.000
	(4482.76)			(1970.61)		
Married	0.624	311		0.585	3222	
School child or younger	0.354	311		0.468	3222	0.000
Education (ref. basic)						
Intermediate	0.379	311		0.493	3222	0.000
Tertiary	0.514	311		0.348	3222	0.000
Unemployment experience	0.876	311		0.882	3222	0.968
Revenue-reducing events in the wake of COVID-19						
Subject to regulation	0.457	311				
Supply problems	0.122	311				
Demand problems	0.434	311				

The table displays mean and standard deviations, in parentheses, for self-employed and gainfully employed individuals

**Table 7:** Summary statistics for self-employed individuals

	(1)	(2)		(3)	(4)	(5)
	Female	Individuals		Male	Individuals	*P*-value of (1)–(3)
Income (gross) decrease	0.632	155		0.471	155	0.004
Working hour decrease	0.536	153		0.455	156	0.156
Remote work	0.432	155		0.481	156	0.392
Demographics						
Age	52.245	155		55.327	156	0.015
	(10.230)			(11.835)		
Female	1.000	155		0.000	156	.
Migration background	0.155	155		0.173	156	0.665
Personality traits						
Openness to experience	0.232	155		0.403	156	0.135
	(1.015)			(1.001)		
Conscientiousness	0.144	155		0.055	156	0.397
	(0.939)			(0.918)		
Extraversion	0.235	155		− 0.050	156	0.009
	(0.835)			(1.066)		
Agreeableness	0.199	155		− 0.207	156	0.000
	(0.941)			(1.036)		
Neuroticism	0.042	155		− 0.296	156	0.002
	(0.970)			(0.910)		
Household context						
Household size	2.626	155		2.609	156	0.917
	(1.378)			(1.479)		
Household net income (€)	4374.67	155		4862.82	156	0.338
	(5021.36)			(3875.48)		
Married	0.613	155		0.635	156	
School child or younger	0.355	155		0.353	156	0.967
Education (ref. basic)						
Intermediate	0.413	155		0.346	156	0.226
Tertiary	0.484	155		0.545	156	0.283
Unemployment experience	0.868	155		0.883	156	0.965
Revenue-reducing events in the wake of COVID-19						
Subject to regulation	0.561	155		0.353	156	0.000
Supply problems	0.110	155		0.135	156	0.504
Demand problems	0.458	155		0.410	156	0.397

The table displays mean and standard deviations, in parentheses, for self-employed individuals

**Table 8:** Summary statistics for employees

	(1)	(2)		(3)	(4)	(5)
	Female	Individuals		Male	Individuals	*P*-value of (1)–(3)
Income (gross) decrease	0.123	1969		0.146	1252	0.063
Working hour decrease	0.205	1959		0.182	1250	0.121
Remote work	0.369	1970		0.423	1252	0.002
Demographics						
Age	47.141	1970		46.866	1252	0.470
	(10.063)			(11.235)		
Female	1.000	1970		0.000	1252	.
Migration background	0.197	1970		0.216	1252	0.193
Personality traits						
Openness to experience	− 0.082	1970		0.046	1252	0.000
	(0.993)			(0.942)		
Conscientiousness	0.164	1970		− 0.063	1252	0.000
	(0.904)			(0.925)		
Extraversion	0.110	1970		− 0.136	1252	0.000
	(1.002)			(1.026)		
Agreeableness	0.036	1970		− 0.282	1252	0.000
	(0.965)			(0.997)		
Neuroticism	0.100	1970		− 0.289	1252	0.000
	(0.985)			(0.905)		
Household context						
Household size	2.875	1970		2.720	1252	0.002
	(1.354)			(1.432)		
Household net income (€)	3763.45	1970		3926.69	1252	0.022
	(1936.66)			(2019.63)		
Married	0.580	1970		0.593	1252	
School child or younger	0.491	1970		0.431	1252	0.001
Education (ref. basic)						
Intermediate	0.535	1970		0.427	1252	0.000
Tertiary	0.327	1970		0.382	1252	0.001
Unemployment experience	0.985	1970		0.719	1252	0.004

The table displays mean and standard deviations, in parentheses, for gainfully employed individuals

**Table 9:** Relevance of industry fixed effects in Table 1

		(1)	(2)	(3)
		Income	Working hours	Remote work
Model without industry fixed effects	Self-employed	0.434***	0.316***	0.014
		(0.029)	(0.030)	(0.031)
	*R*^2^	0.12	0.07	0.21
Unrestricted model	Self-employed	0.421***	0.302***	0.021
		(0.031)	(0.031)	(0.032)
	*R*^2^	0.23	0.13	0.31

The table displays the coefficient estimates and *R*-squared of the unrestricted models in columns (2), (4), and (6) of Table 1 with and without the inclusion of industry fixed effects. Corresponding robust standard errors are in parentheses. **p* < 0.10, ***p* < 0.05, ****p* < 0.01

**Table 10:** Detailed results for the Gelbach decomposition of the gender gap among self-employed individuals

	(1)	(2)	(3)
	Income	Working hours	Remote work
Total change	0.093*	0.119**	0.022
	(0.049)	(0.049)	(0.051)
Demographics	0.031*	0.007	0.018
	(0.017)	(0.014)	(0.018)
NACE	0.092**	0.121***	0.000
	(0.045)	(0.043)	(0.041)
Big 5	− 0.029	− 0.010	− 0.005
	(0.023)	(0.026)	(0.024)
Household context	− 0.001	− 0.003	0.016
	(0.012)	(0.014)	(0.016)
Unemployment experience	− 0.002	0.000	0.001
	(0.007)	(0.001)	(0.004)
Education	0.001	0.004	− 0.008
	(0.006)	(0.006)	(0.010)

The table displays the detailed results of the Gelbach decomposition of the gender gap among self-employed individuals. Columns (1), (2), and (3) display the results for the likelihood of an income decline, decline in working hours and working from home. The total change corresponds to the change in the gender gap between the restricted and the unrestricted models. The remaining rows show the contribution of the respective groups of covariates to the total change. Corresponding robust standard errors are in parentheses. **p* < 0.10, ***p* < 0.05, ****p* < 0.01

**Table 11:** Restricted and unrestricted models for differences in the likelihood that income or working hours decreased or that individuals transitioned into non-employment between employees and self-employed respondents, conditional on the employment status in 2019

	(1)	(2)	(3)	(4)	(5)	(6)
	Income	Income	Working	Working	Job	Job
			hours	hours	loss	loss
Self-employed	0.366***	0.364***	0.266***	0.267***	0.012	− 0.007
	(0.031)	(0.033)	(0.031)	(0.033)	(0.009)	(0.018)
Demographics						
Gender: female		0.015		0.021		0.007
		(0.014)		(0.016)		(0.005)
Age		0.001		− 0.003		− 0.003*
		(0.005)		(0.006)		(0.002)
Age squared		0.000		0.000		0.000*
		(0.000)		(0.000)		(0.000)
Migration background		0.037**		0.042**		0.008
		(0.017)		(0.020)		(0.007)
Big 5						
Extraversion		0.005		0.011		0.005**
		(0.006)		(0.007)		(0.002)
Conscientiousness		− 0.008		− 0.022***		− 0.001
		(0.007)		(0.008)		(0.002)
Openness to experience		0.010		0.005		0.002
		(0.006)		(0.008)		(0.002)
Neuroticism		− 0.003		0.002		0.002
		(0.006)		(0.008)		(0.002)
Agreeableness		0.001		− 0.005		0.003
		(0.006)		(0.007)		(0.002)
Household context						
HH size		0.009		0.015*		0.001
		(0.008)		(0.009)		(0.003)
Married		0.015		0.014		0.005
		(0.016)		(0.018)		(0.006)
School child or younger		0.014		− 0.005		0.000
		(0.019)		(0.021)		(0.007)
Log of HH net income		− 0.044***		− 0.042**		− 0.009
		(0.017)		(0.019)		(0.006)
Education (ref. low)						
Intermediate education		0.045**		0.023		− 0.006
		(0.019)		(0.023)		(0.008)
High education		0.031		0.001		− 0.001
		(0.022)		(0.025)		(0.009)
Unemployment experience		0.000		0.007*		0.004**
		(0.003)		(0.003)		(0.002)
Mean of outcome	0.168	0.168	0.219	0.219	0.017	0.017
Observations	3348	3348	3334	3334	3661	3661
*R*^2^	0.08	0.22	0.04	0.13	0.01	0.05

The table displays models with and without controls for differences between self-employed and employees. All models include state and week fixed effects. Columns (1), (3), and (5) display results for the models without controls. Columns (2), (4), and (6) display results for the models with controls. The unrestricted models also include NACE 2 fixed effects. Standard errors are robust and in parentheses. **p* < 0.10, ***p* < 0.05, ****p* < 0.01

**Table 12:** The share of women and industry fixed effects for income losses

	Rank	NACE code	Description	Share female	FE estimate
	(1)	(2)	(3)	(4)	(5)
High share women	1	96	Other personal service activities	0.857	0.480**
					(0.236)
	2	88	Social work activities without accommodation	0.832	0.124
					(0.242)
	3	47	Retail trade, except of motor vehicles and motorcycles	0.818	0.775***
					(0.222)
	4	55	Accommodation	0.818	0.283
					(0.242)
	5	86	Human health activities	0.803	0.405*
					(0.208)
Low share women	1	49	Land transport and transport via pipelines	0.189	0.463
					(0.334)
	2	18	Printing and reproduction of recorded media	0.235	− 0.425*
					(0.234)
	3	43	Specialized construction activities	0.273	0.093
					(0.249)
	4	62	Computer programming, consultancy and related activities	0.290	0.098
					(0.246)
	5	28	Manufacture of machinery and equipment n.e.c.	0.297	0.738***
					(0.218)

The table displays the share of women and the associated income loss fixed effects for the industries with the highest share and lowest share of women. For the table, we display only industries with at least ten observations. Column (1) displays the rank within each panel. Columns (2) and (3) display the two-digit NACE code and the description, respectively. Column (3) displays the share of women within each occupation in our full sample. Column (5) displays industry fixed effect estimates, which stem from a regression of the likelihood of an income loss on state and week indicators as well as industry indicators, along with robust standard errors in parentheses. The reference industry is “Crop and animal production, hunting and related service activities.” **p* < 0.10, ***p* < 0.05, ****p* < 0.01

**Table 13:** Restricted and unrestricted models for the likelihood that income and working hours decreased or individuals are working from home among employees

	(1)	(2)	(3)	(4)	(5)	(6)
	Income	Income	Working hours	Working hours	Remote work	Remote work
Gender: female	− 0.022*	0.014	0.021	0.026	− 0.048***	− 0.009
	(0.012)	(0.013)	(0.014)	(0.016)	(0.018)	(0.018)
Demographics						
Age		− 0.004		− 0.008		0.000
		(0.005)		(0.006)		(0.006)
Age squared		0.000		0.000		0.000
		(0.000)		(0.000)		(0.000)
Migration background		0.041**		0.031		− 0.020
		(0.016)		(0.019)		(0.019)
Big 5						
Extraversion		− 0.002		0.005		− 0.007
		(0.006)		(0.007)		(0.008)
Conscientiousness		0.007		− 0.014*		− 0.003
		(0.006)		(0.008)		(0.008)
Openness to experience		− 0.010		0.002		0.026***
		(0.007)		(0.008)		(0.008)
Neuroticism		− 0.005		− 0.002		− 0.009
		(0.006)		(0.008)		(0.008)
Agreeableness		0.000		− 0.005		0.005
		(0.006)		(0.007)		(0.008)
Household context						
HH size		0.009		0.016*		− 0.019**
		(0.007)		(0.008)		(0.009)
Married		0.021		0.027		− 0.031*
		(0.015)		(0.018)		(0.019)
School child or younger		0.014		− 0.014		0.049**
		(0.018)		(0.021)		(0.023)
Log of HH net income		− 0.028*		− 0.044**		0.151***
		(0.016)		(0.019)		(0.020)
Education (ref. low)						
Intermediate education		0.035*		0.016		0.069***
		(0.019)		(0.023)		(0.020)
High education		0.018		− 0.008		0.283***
		(0.021)		(0.025)		(0.025)
Unemployment experience		0.003		0.005*		− 0.002
		(0.003)		(0.003)		(0.002)
Mean of outcome	0.132	0.132	0.196	0.196	0.390	0.390
Observations	3221	3221	3209	3209	3222	3222
*R*^2^	0.01	0.17	0.01	0.10	0.03	0.34

The table displays restricted and unrestricted models underlying the Gelbach decomposition. All models include state and week fixed effects. Columns (1), (3) and (5) display results for the restricted models. Columns (2), (4) and (6) display results for the unrestricted models. The unrestricted models also include NACE 2 fixed effects. Standard errors are robust and in parentheses. **p* < 0.10, ***p* < 0.05, ****p* < 0.01

**Table 14:** Detailed results for the Gelbach decomposition of the gender gap in potential mechanisms among self-employed individuals

	(1)	(2)	(3)
	Restrictions	Supply	Demand
Total change	0.151***	0.030	0.059
	(0.049)	(0.033)	(0.050)
Demographics	0.017	0.022**	0.044**
	(0.015)	(0.011)	(0.018)
NACE	0.089*	0.022	0.021
	(0.046)	(0.028)	(0.043)
Big 5	0.046**	− 0.012	− 0.002
	(0.022)	(0.017)	(0.025)
Household context	0.000	− 0.002	− 0.004
	(0.010)	(0.007)	(0.011)
Unemployment experience	0.002	0.001	0.002
	(0.006)	(0.003)	(0.007)
Education	− 0.003	− 0.002	− 0.001
	(0.005)	(0.006)	(0.005)

The table displays the detailed results of the Gelbach decomposition of the gender gap in potential mechanisms among self-employed individuals. Columns (1), (2), and (3) display the results for the likelihood of an income decline, decline in working hours and working from home. The total change corresponds to the change in the gender gap between the restricted and the unrestricted models. The remaining rows show the contribution of the respective groups of covariates to the total change. Corresponding robust standard errors are in parentheses. **p* < 0.10, ***p* < 0.05, ****p* < 0.01

**Table 15:** Detailed results for the Gelbach decomposition of the gender gap in the likelihood of an income decline among self-employed individuals, including business-related events as an explanatory variable

	(1)	(2)	(3)
	Restrictions	Supply	Demand
Total change	0.103**	0.092*	0.089*
	(0.049)	(0.049)	(0.051)
NACE	0.071*	0.091**	0.083**
	(0.043)	(0.045)	(0.042)
Event	0.045**	− 0.001	0.020
	(0.018)	(0.003)	(0.022)
Remainder	− 0.013	0.001	− 0.013
	(0.029)	(0.030)	(0.025)

The table displays the detailed results of the Gelbach decomposition of the gender gap in the likelihood of an income decline among self-employed individuals. Columns (1), (2), and (3) display the results including and indicator whether respondents state their business has been affected by restrictions or policies, supply or demand shortages in the wake of the COVID-19 pandemic, respectively. The total change corresponds to the change in the gender gap between the restricted and the unrestricted models. The remaining characteristics are included in the group “Remainder.” Corresponding robust standard errors are in parentheses. **p* < 0.10, ***p* < 0.05, ****p*< 0.01

## Appendix 2. Derivation of the Gelbach decomposition

Assume two sets of variables, *X*_1_ and *X*_2_, with *k*_1_ and *k*_2_ variables each.^28^ The population linear relationship is given by:

1 $$ Y = X_{1} \beta_{1} + X_{2} \beta_{2} + \epsilon $$

We label the components of the OLS estimator that correspond to the variables in *X*_1_ and *X*_2_, $\hat {\beta }_{1}^{full}$ and $\hat {\beta }_{2}$, respectively.

Thus, we obtain:

2 $$ y = X_{1}\hat{\beta}_{1}^{full} + X_{2} \hat{\beta}_{2} + \hat\epsilon $$

Now let us consider the coefficient on *X*_1_ from a base specification that completely ignores the variables in *X*_2_. We denote this estimator $\hat {\beta }_{1}^{base} = (X_{1}^{\intercal } X_{1})^{-1} X_{1}^{\intercal } y$.

The Gelbach (2016) decomposition answers the question of how much of the change in *X*_1_ coefficients can be attributed to different variables in *X*_2_ as we move from the base specification that has no *X*_2_ covariates to the full specification that includes both *X*_1_ and all *X*_2_ covariates. In the context of our analysis, *X*_1_ would refer to a gender indicator, and *X*_2_ to the full set of control variables. Throughout the analysis, we partial out week and state fixed effects. The decomposition links the estimates of the base and full specification on *X*_1_ through the following identity, which is obtained by pre-multiplying both sides of Eq. 2 by $(X_{1}^{\intercal } X_{1})^{-1} X_{1}^{\intercal }$ and using the orthogonality of the fitted residuals to the columns of *X*_1_:

3 $$ \hat{\beta}_{1}^{base} = \hat{\beta}_{1}^{full} + (X_{1}^{\intercal} X_{1})^{-1} X_{1}^{\intercal} X_{2} \hat{\beta}_{2} $$

Re-writing the above identity and defining the change in the coefficient on the gender dummy between the base and the full model as $\hat {\delta } \equiv \hat {\beta }_{1}^{base} - \hat {\beta }_{1}^{full}$, one obtains

4 $$ \hat{\delta} \equiv \hat{\beta}_{1}^{base} - \hat{\beta}_{1}^{full} = (X_{1}^{\intercal} X_{1})^{-1} X_{1}^{\intercal} X_{2} \hat{\beta}_{2}, $$

which corresponds to the omitted variable bias formula.

Let *X*_2*k*_ be the column of observations on the *k* th covariate in *X*_2_ and let $\hat {\beta }_{2k}$ be the estimated coefficient on *X*_2*k*_ in the full specification, then

5 $$ \hat{\delta} = \sum\limits_{k=1}^{k_{2}} (X_{1}^{\intercal} X_{1})^{-1} X_{1}^{\intercal} X_{2k} \hat{\beta}_{2k}, $$

since the omitted variables bias formula is linear in its *k*_2_ components.

From there, the practical implementation of the decomposition follows naturally:

1. Estimate the full model to obtain $\hat {\beta }_{2}$.
2. Estimate the vector of coefficients on *X*_1_ in a set of OLS regressions with each of the *k*_2_ covariates *X*_2*k*_ as dependent variable. This yields $(X_{1}^{\intercal } X_{1})^{-1} X_{1}^{\intercal } X_{2k}$.
3. Multiply $(X_{1}^{\intercal } X_{1})^{-1} X_{1}^{\intercal } X_{2k}$ by $\hat {\beta }_{2k}$ to obtain $\hat {\delta _{k}}$, which is the component estimated to be due to each variable *k*.

The set of covariates we include in our Gelbach decomposition, i.e., *X*_2_, are:

- Demographics: second-order polynomial in age, indicator for a migration background,
- NACE codes (2019): indicators for the two-digit NACE codes,
- Big 5 (2019): openness to experience, conscientiousness, extraversion, agreeableness, and neuroticism,
- Household context (2019): household size, indicators for being married, presence of school children (or younger) in the household, the logarithm of household net income (2019/18) and
- unemployment experience (2018).

## Footnotes

1 Under this scheme, employers send their employees into short-time work where the Federal Employment Office subsidizes a large portion of the wage costs pertaining to those contractual working hours that employees are not working. This instrument allows employers to keep their workforce through the crisis while protecting employees from losing their jobs, and from major wage losses, see also Cahuc (2019).

2 There are also various studies investigating the effects of the COVID-19 pandemic on overall employment (Forsythe et al. 2020; Chetty et al. 2020; Cajner et al. 2020; Juranek et al. 2020; Coibion et al. 2020; Adams-Prassl et al. 2020).

3 We use the SOEPv35. DOI: 10.5684/soep-core.v35. In addition, we use the preliminary data of the SOEP for 2019.

4 See Table 11 in Appendix 1 and Section 4.1 for a discussion of job loss due to the pandemic.

5 Note that the question on the precise amount of income losses was not included in the first of the nine waves of the SOEP-COV. Since this happens to be the wave with the largest number of interviewees, we are left with 104 self-employed individuals who reported income losses. We report both median and mean losses, but consider the median a superior statistic of centrality in this context, given that the distribution of income losses is, as expected, strongly right skewed (Sorgner et al. 2017).

6 Compared to the previous analysis, we lose 23 observations. The final sample includes 81 observations. Moreover, we do not adjust for inflation. However, first projections indicate that the inflation rate is approximately close to zero for 2020. Lastly, we partly observe individuals in different months, i.e., we are not able to account for seasonality. This possibly introduces some additional measurement error.

7 We have information on reductions in working hours for all waves of the SOEP-CoV.

8 For the figures, we dropped a single observation with a relative reduction of 10.

9 Note that the estimates displayed in odd columns somewhat differ from the raw self-employment gap due to the inclusion of state and time fixed effects. The inclusion of these fixed effects is important since individuals in the sample were interviewed at different stages of the pandemic. Over time, the incidence of, for example, working from home changed. Thus, an accurate reflection of a self-employment (gender) gap requires that the dynamic of the pandemic is accounted for.

10 Once industry effects are added, the *R*-squared increases by around 50% for the probability of working from home while nearly doubling for the probability of facing a decrease in income and hours, respectively.

11 For a more detailed analysis on the differences in the estimates of industry fixed effects, see Graeber et al. (2021).

12 Once again, the estimates displayed in the restricted models somewhat differ from the raw gender gap due to the inclusion of state and time fixed effects.

13 The corresponding analysis of the magnitude of earnings losses are shown in Graeber et al. (2021). Since sample sizes decrease considerably, the analysis suffers from imprecision. Effect sizes still confirm our main conclusions, even for the changes at the intensive margin.

14 Detailed results of the Gelbach decomposition are depicted in Table 10 in Appendix 1.

15 In Fig. 7, we calculate the share of women in the respective industries over the complete working sample, i.e. we do not distinguish between self-employed and employed individuals.

16 Note that the possibility to work from home has a moderating effect on the likelihood of an income and working hours decline. If we include the indicator for working from home in the unrestricted models, the coefficients suggest that working from home is associated with a 13.72 (*p* = 0.063) and 15.62 (*p* = 0.041) percentage point lower likelihood of a decline in income or working hours, respectively. However, the Gelbach decomposition suggests that working from home because of the COVID-19 pandemic does not contribute to the gender difference in these two outcomes. These results are available upon request.

17 There might also exist initial gender differences in the standard workload among the self-employed. Therefore, we also examined whether including actual weekly working hours of the previous year alters the estimated gender gaps in a meaningful way, which is not the case.

18 In our sample, the industries with the highest shares of women are, in decreasing order, “Other personal service activities,” “Social work activities without accommodation,” “Retail trade, except of motor vehicles and motorcycles,” “Accommodation,” and “Human health activities.” A detailed breakdown of industries is limited by sample size restrictions. In Table 12 in Appendix 1, we only display industries with at least ten observations.

19 The reference category is the agricultural sector.

20 The five industries with the lowest shares of females are, in increasing order, “Land transport and transport via pipelines,” “Printing and reproduction of recorded media,” “Specialized construction activities,” “Computer programming, consultancy and related activities,” and “Manufacture of machinery and equipment n.e.c.”

21 See Table 6 and Table 7 in Appendix 1 for summary statistics on the dependent variables used in this section.

22 The detailed results for the Gelbach decomposition are depicted in Table 14 in Appendix 1.

23 For the sake of brevity, we consolidate all other characteristics in the category “Remainder.”

24 Detailed results for the Gelbach decomposition are displayed in Table 15 in Appendix 1.

25 At this point, we abstract from gender norms, which could also explain the gendered response to a closure of childcare facilities.

26 We are very grateful to an anonymous referee who suggested the discussion of intra-household dynamics.

27 We abstract from leisure in this analysis since we assume that individuals shift their time from market production to household chores.

## References

[CR1] Adams-Prassl A, Boneva T, Golin M, Rauh C (2020). Inequality in the impact of the coronavirus shock: Evidence from real time surveys. J Public Econ.

[CR2] Alipour, J-V, Falck O, Schüller S (2020) Germany’s capacities to work from home. CESifo Working Paper 8227, Center for Economic Studies and ifo Institute (CESifo)

[CR3] Alon TM, Doepke M, Olmstead-Rumsey J, Tertilt M (2020) The impact of COVID-19 on gender equality. Working Paper 26947, National Bureau of Economic Research

[CR4] Audretsch DB, Kritikos AS, Schiersch A (2020). Microfirms and innovation in the service sector. Small Bus Econ.

[CR5] Barbieri T, Basso G, Scicchitano S (2020) Italian workers at risk during the covid-19 epidemic. GLO Discussion Paper Series 513, Global Labor Organization (GLO)

[CR6] Barro RJ, Ursúa JF, Weng J (2020) The coronavirus and the Great Influenza Pandemic: Lessons from the “Spanish flu” for the coronavirus’s potential effects on mortality and economic activity NBER Working Papers 26866. National Bureau of Economic Research, Inc

[CR7] Beland L-P, Fakorede O, Mikola D (2020) The Short-Term Effect of COVID-19 on Self-Employed Workers in Canada. GLO Discussion Paper Series 585, Global Labor Organization (GLO) 10.3138/cpp.2020-076 PMC7976854 38629981

[CR8] Bertrand M, Kamenica E, Pan J (2015). Gender identity and relative income within households. Q J Econ.

[CR9] Blau FD, Kahn LM (2017). The gender wage gap: Extent, trends, and explanations. J Econ Lit.

[CR10] Block J, Kritikos AS, Priem M, Stiel C (2020) Emergency aid for self-employed in the COVID-19 pandemic: A flash in the pan? DIW Discussion Paper 1924. DIW Berlin, German Institute for Economic Research 10.1016/j.joep.2022.102567 PMC9547119 36245552

[CR11] Blundell J, Machin S (2020) Self-employment in the Covid-19 crisis CEP Covid-19 Briefings cepcovid-19-003. Centre for Economic Performance, LSE

[CR12] Bonacini L, Gallo G, Patriarca F (2021). Identifying policy challenges of COVID-19 in hardly reliable data and judging the success of lockdown measures. J Popul Econ.

[CR13] Bönte W, Piegeler M (2013). Gender gap in latent and nascent entrepreneurship: driven by competitiveness. Small Bus Econ.

[CR14] Burda MC (2016) The German labor market miracle, 2003-2015: An assessment. SFB 649 Discussion Paper 2016-005, Humboldt University of Berlin, Collaborative Research Center 649 - Economic Risk

[CR15] Cahuc P (2019) Short-time work compensation schemes and employment. IZA World of Labor, 1–11

[CR16] Cajner T, Crane LD, Decker RA, Grigsby J, Hamins-Puertolas A, Hurst E, Kurz C, Yildirmaz A (2020) The U.S. Labor Market during the Beginning of the Pandemic Recession NBER Working Papers 27159. National Bureau of Economic Research, Inc

[CR17] Caliendo M, Fossen FM, Kritikos AS, Wetter M (2014). The gender gap in entrepreneurship: Not just a matter of personality. CESifo Econ Stud.

[CR18] Caliendo M, Künn S (2015). Getting back into the labor market: The effects of start-up subsidies for unemployed females. J Popul Econ.

[CR19] Chetty R, Friedman JN, Hendren N, Stepner M, Team TOI (2020) The economic impacts of COVID-19: Evidence from a new public database built using private sector data NBER Working Papers 27431. National Bureau of Economic Research, Inc 10.1093/qje/qjad048 PMC11189622 38911676

[CR20] Coibion O, Gorodnichenko Y, Weber M (2020) Labor Markets During the COVID-19 Crisis: A Preliminary View NBER Working Papers 27017. National Bureau of Economic Research, Inc

[CR21] Correia S, Luck S, Verner E (2020) Pandemics depress the economy, public health interventions do not: Evidence from the 1918 flu. SSRN Electron J

[CR22] Davidsson P, Gordon SR (2016). Much ado about nothing? The surprising persistence of nascent entrepreneurs through macroeconomic crisis. Entrep Theory Pract.

[CR23] Dingel J, Neiman B (2020). How many jobs can be done at home?. J Public Econ.

[CR24] Doern R (2016). Entrepreneurship and crisis management: The experiences of small businesses during the London 2011 riots. Int Small Bus J.

[CR25] Doern R, Williams N, Vorley T (2019). Special issue on entrepreneurship and crises: business as usual? An introduction and review of the literature. Entrepreneurship Reg Dev.

[CR26] Elam AB, Brush CG, Greene PG, Baumer B, Dean M, Heavlow R (2019) Global entrepreneurship monitor: 2018/2019 women’s entrepreneurship report. Technical report, Global Entrepreneurship Research Association

[CR27] Fairlie RW (2020). The impact of COVID-19 on small business owners: Evidence from the first three months after widespread social-distancing restrictions. J Econ Manag Strat.

[CR28] Federal Ministry for Economic Affairs and Energy (2020) German government announces €50 billion in emergency aid for small businesses. https://www.bmwi.de/Redaktion/EN/Pressemitteilungen/2020/20200323-50-german-government-announces-50-billion-euros-in-emergency-aid-for-small-businesses.html, accessed 2020-10-05

[CR29] Federal Ministry of Health (2020) Coronavirus SARS-CoV-2: Chronik der bisherigen maßnahmen. https://www.bundesgesundheitsministerium.de/coronavirus/chronik-coronavirus.html, accessed 2020-10-21

[CR30] Forsythe E, Kahn LB, Lange F, Wiczer D (2020). Labor demand in the time of COVID-19: Evidence from vacancy postings and UI claims. J Public Econ.

[CR31] Foster G, Stratton LS (2021). Does female breadwinning make partnerships less healthy or less stable?. J Popul Econ.

[CR32] Fritsch M, Kritikos AS, Sorgner A (2015). Why did self-employment increase so strongly in Germany?. Entrepreneurship Reg Dev.

[CR33] Gelbach JB (2016). When do covariates matter? And which ones, and how much?. J Labor Econ.

[CR34] Georgellis Y, Wall HJ (2005). Gender differences in self-employment. Int Rev Appl Econ.

[CR35] Goebel J, Grabka MM, Liebig S, Kroh M, Richter D, Schröder C, Schupp J (2019). The German Socio-Economic Panel (SOEP). Jahrbü,cher für Nationalökonomie und Statistik.

[CR36] Goldin C, Kerr SP, Olivetti C, Barth E (2017). The expanding gender earnings gap: Evidence from the LEHD-2000 census. Am Econ Rev Papers Proc.

[CR37] Graeber D, Kritikos AS, Seebauer J (2021) COVID-19: a crisis of the female self-employed. GLO Discussion Paper Series 783, Global Labor Organization (GLO) 10.1007/s00148-021-00849-y PMC8192686 34131364

[CR38] Günther L, Marder-Puch K (2019) Selbstständigkeit-Methoden und Ergebnisse des Ad-hoc-Moduls zur Arbeitskräfteerhebung 2017. Wirtschaft und Statistik (WISTA) 1

[CR39] Ifo Institute and forsa (2020) Erste Ergebnisse des Befragungsteils der BMG-Corona-BUND-Studie. https://www.ifo.de/DocDL/bmg-corona-bund-studie-erste-ergebnisse.pdf, accessed 2020-10-05.

[CR40] Juranek S, Paetzold J, Winner H, Zoutman F (2020) Labor market effects of COVID-19 in Sweden and its neighbors: Evidence from novel administrative data. Discussion Paper 2020/8, NHH Dept. of Business and Management Science

[CR41] Kalenkoski CM, Pabilonia SW (2020) Initial impact of the COVID-19 pandemic on the employment and hours of self-employed coupled and single workers by gender and parental status. IZA Discussion Paper 13443, IZA – Institute of Labor Economics

[CR42] Karlsson M, Nilsson T, Pichler S (2014). The impact of the 1918 Spanish flu epidemic on economic performance in Sweden: An investigation into the consequences of an extraordinary mortality shock. J Health Econ.

[CR43] Kühne S, Kroh M, Liebig S, Zinn S (2020). The need for household panel surveys in times of crisis: The case of soep-cov. Surv Res Methods.

[CR44] Koellinger P, Minniti M, Schade C (2013). Gender differences in entrepreneurial propensity. Oxf Bull Econ Stat.

[CR45] Kritikos AS, Graeber D, Seebauer J (2020) Corona-Pandemie wird zur Krise für Selbständige. DIW aktuell 47

[CR46] Leoni T, Falk M (2010). Gender and field of study as determinants of self-employment. Small Bus Econ.

[CR47] Meara K, Pastore F, Webster A (2020). The gender pay gap in the USA: a matching study. J Popul Econ.

[CR48] Milani F (2021). Covid-19 outbreak, social response, and early economic effects: a global VAR analysis of cross-country interdependencies. J Popul Econ.

[CR49] OECD (2017) Entrepreneurship at a glance. https://www.oecd-ilibrary.org/docserver/entrepreneur_aag-2017-en.pdf?expires=1610536834&id=id&accname=guest&checksum=D90265D7A9E5AB441BB39E57CA65F57A, accessed 2021-13-01

[CR50] Parker SC (2018). The economics of entrepreneurship.

[CR51] Patrick C, Stephens H, Weinstein A (2016). Where are all the self-employed women? Push and pull factors influencing female labor market decisions. Small Bus Econ.

[CR52] Petrovan, S., Aldridge D, Bartlett H, Bladon A, Booth H, Broad S, Broom D, Burgess N, Cunningham A, Ferri M, Hinsley A, Hughes A, Jones K, Kelly M, Mayes G, Ugwu C, Uddin N, Verissimo D, White T, Sutherland W (2020) Post COVID-19: a solution scan of options for preventing future zoonotic epidemics 10.1111/brv.12774 PMC8444924 34231315

[CR53] Qiu Y, Chen X, Shi W (2020). Impacts of social and economic factors on the transmission of coronavirus disease 2019 (COVID-19) in China. J Popul Econ.

[CR54] Siemer M (2014) Firm entry and employment dynamics in the great recession. Finance and Economics Discussion Series 2014-56, Board of Governors of the Federal Reserve System (U.S.)

[CR55] Sorgner A, Fritsch M, Kritikos AS (2017). Do entrepreneurs really earn less?. Small Bus Econ.

[CR56] Stoica O, Roman A, Rusu VD (2020). The nexus between entrepreneurship and economic growth: A comparative analysis on groups of countries. Sustainability.

[CR57] Velde FR (2020) What happened to the US economy during the 1918 Influenza pandemic? A view through high-frequency data. Working Paper WP 2020-11, Federal Reserve Bank of Chicago

[CR58] von Gaudecker H-M, Holler R, Janys L, Siflinger BM, Zimpelmann C (2020) Labour supply in the early stages of the COVID-19 pandemic: Empirical evidence on hours, home office, and expectations. IZA Discussion Papers 13158, Institute of Labor Economics (IZA)

[CR59] Weiss Y (1993) The formation and dissolution of families: Why marry? Who marries whom? And what happens upon divorce. In: Rosenzweig MR, Stark O (eds) Handbook of population and family economics, Volume 1 of Handbook of Population and Family Economics, Chapter 3. Elsevier, pp 81–123

